# Inhibitions imposed by kinetic constraints of membranes in all-solid-state ion-selective electrodes: characteristics of interfacial capacitance in solid contacts†

**DOI:** 10.1039/d5sc01241d

**Published:** 2025-04-30

**Authors:** Rui-Ze Xia, Xin Cai, Jing-Yi Lin, Yong-Huan Zhao, Zi-Hao Liu, Chen-Lu Wang, Shi-Hua Chen, Meng Yang, Zong-Yin Song, Pei-Hua Li, Xing-Jiu Huang

**Affiliations:** a Key Laboratory of Environmental Optics and Technology, Environmental Materials and Pollution Control Laboratory, Institute of Solid State Physics, HFIPS, Chinese Academy of Sciences Hefei 230031 China chenshh@issp.ac.cn myang@iim.ac.cn zysong@issp.ac.cn peihuali@issp.ac.cn xingjiuhuang@iim.ac.cn; b Department of Materials Science and Engineering, University of Science and Technology of China Hefei 230026 China

## Abstract

Although various materials have been extensively studied as solid contacts in all-solid-state ion-selective electrodes, of research on kinetic phenomena at solid–solid and solid–liquid interfaces remains limited. This lack of understanding may lead to confusion between the performance of capacitors and that of electrical analysis systems, finally leading to misinterpretation of material properties. While there are established methodologies for investigating capacitive mechanisms, they all center on the energy storage properties of particular materials and lack the capability to analyze real detection systems involving membranes. This study proposes an algorithm to investigate electrode interfaces with complex structures and uncovers the impact of membranes on the capacitance of solid contacts through experimental data and simulations. Electrochemical impedance spectroscopy is clustered using a machine learning algorithm and then analysis of the distribution of relaxation times is utilized to simulate results and generate multiple models for electrode interfaces. Step potential electrochemical spectroscopy is simulated based on the electrode interface model to quantitatively analyze specific charge storage processes. Simulated results revealed that the symmetry of primary charge processes under varying overpotentials for different solid contacts is proportional to the conversion ratios of the capacitance of each material, which is attributed to inhibition on the electrode interfaces of ion-selective membranes. This work highlights the importance of considering interactions between membranes and materials in the development of transduction materials and can also be extended to investigate electrode interfaces, not only all-solid-state ion-selective electrodes.

## Abstract 2

This study combines electrochemistry and interface analysis to reveal how ion-selective membranes limit transduction layer capacitance in all-solid-state ISEs, guiding material selection and performance evaluation.

## Graphical abstract

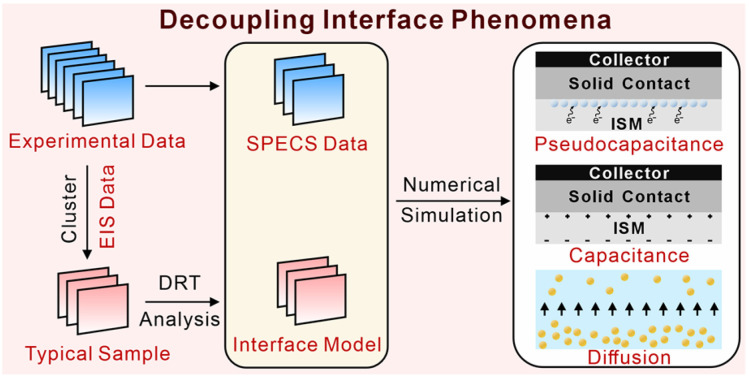

## 1 Introduction

Electrolytes in the body can serve as a valuable indicator for clinical diagnosis and sports medicine,^1^ but real-time monitoring is challenging due to the size of the equipment and the high costs involved.^2^ The trend towards miniaturization and flexibility of sensors has led to solid contact ion-selective electrodes (SCISE) showing great potential in the portable detection of human fluid electrolytes.^3^ Nowadays, there is a proliferation of solid contact materials with high hydrophobicity and large capacitance for all-solid-state potentiometric sensors,^4–6^ which lays a solid foundation for the construction of highly stable ion-sensitive recognition interfaces. In terms of wearable devices, Gao *et al.* exploited a flexible, wearable system utilizing conducting polymer to measure and analyse human physiological states, thereby advancing the development of real-time detection devices.^7^ Cai *et al.* designed a wristwatch with a fully integrated structure, incorporating modified graphene materials as the solid contact to achieve reliable detection of human electrolytes outside laboratory settings.^8^ Although many researchers have made the significant advancements, mentioned above, by focusing on front-end system design and solid contact properties, such as capacitance and hydrophobicity,^9,10^ the relationships between different functional components have sometimes been overlooked. Recently, Chipangura *et al.* proposed that the influence of membrane compositions on solid contacts should be adopted as one important reference criterion for the design of solid contact materials.^11^ A full understanding of the dynamic interface phenomena that occur at the interface can help researchers improve sensor performance and guide the development of materials.

The most commonly used method to investigate interfacial capacitive properties is *b*-value analysis, which empirically describes the relationship between response currents and scan rates in cyclic voltammetry.^12^ By using specific *b*-values, it is possible to elucidate the mechanism of the material. The mechanisms can be divided into three types: capacitive (pure capacitance), pseudocapacitive (involving redox reactions and intercalation on surfaces), and redox-capacitive (related to diffusion phenomena).^13,14^ Liu *et al.* utilized cyclic voltammetry and X-ray adsorption spectroscopy to examine pseudocapacitive solid contacts, which analysed the fine structural changes in materials but did not provide information about material–membrane interfaces.^15^ Dai *et al.* also used the *b*-value method to demonstrate surface-controlled solid contact (*b* ≈ 1), while inner diffusion and solution diffusion were not distinguishable when *b* < 1, which may lead to inadequate understanding of mechanisms due to significant changes in the crystal structures of SnS_2_–MoS_2_, with the disappearance of internal diffusion.^16^ However, the lack of physical explanations for *b*-values implies that this method cannot distinguish capacitance from pseudo-capacitance, or diffusion occurring in solutions from diffusion within materials.^17^ As a result, many critiques have been proposed and some improvements to the *b*-value method have also been suggested.^18–21^

Recently, Bergh *et al.* proposed an impressive scheme that extended the concept of *b*-values from discrete values to continuous differentiation. This study elucidated how specific factors, such as film thickness, electrolyte concentration, and nanoscale dimensions, impact a series of *b*-values and explored the structure–function relationships between materials and various forms of diffusion.^22^ This study is of great value, but it is still not feasible to unravel the interconnected complex interface processes nor can it be achieved for multi-interface systems. There are additional electrochemical methods, such as 3D-electrochemical impedance spectroscopy (3D-EIS) and step potential electrochemical spectroscopy (SPECS).^23–26^ The former cannot guarantee specific physical meanings of fitted models, while the latter may result in deviations from the actual electrochemical system due to the complexity of global optimization. Another significant issue is that these methods are specifically designed for energy storage devices, rendering them inapplicable for SCISEs. Electrochemical systems in those methods often include modified electrodes immersed in solutions with target ions without membranes, which leads to a gap between the theoretical expectation of sensor designs and the performance of real devices; thus, the explanation of electroanalytical signals seems to be uncertain. Although Zdrachek *et al.* suggested that cyclic voltammetry should be conducted in organic solvents to mimic real environments, significant differences remain in physical and chemical properties compared to SCISEs.^27^ The limitation of complex interface analysis methods directly results in ambiguity about the correlation between parameters like selectivity, long-term stability, detection limit, and transduction layer materials in practical applications, which is disadvantageous to the development of transduction layer materials and the transformation of experimental results. As the drawbacks of these methods have already been discussed, it is evident that an algorithm that can deal with complex interfacial systems is necessary, especially for SCISEs.

This study utilized an integrated algorithm to quantitatively analyse the chemical and physical processes taking place on electrode interfaces, as well as to investigate limitations on the capacitance of solid contacts caused by membranes. Machine learning was employed to classify origin EIS data into clusters, followed by analysis of the distribution of relaxation times (DRT). The interface models identified through DRT analyses were input into SPECS simulations to calculate interfacial processes quantitatively. Through the identification of specific charge processes, correlations between the symmetry of charge storage processes at varying overpotentials and the capacitive conversion ratio were observed. More importantly, the presence of ion-selective membranes was found to limit the capacitance of different materials, leading to a significant disparity in the capacitance of solid contacts in operational detection states compared to modified electrodes without membranes. This indicates that, instead of solely prioritizing the large capacitance of capacitors, it is equally crucial to emphasize the interactions between materials and membranes.

## 2 Experiment and theory

### 2.1 Experimental section

The electrochemical system consists of a bare glassy carbon electrode (GCE) with a radius of 0.15 cm (geometric area of 7.07 × 10^−2^ cm^2^), a platinum wire counter electrode, and an Ag/AgCl reference electrode. All experiments were conducted using six GCEs, and a unified cleaning program was executed before loading different materials for measurements. A 0.1 M NaCl solution was used as the electrolyte solution, necessitating the use of an ion-selective membrane with sodium ion carriers. The study comparatively investigates electrode interface processes by analysing measurable differences induced either through varied transduction materials with fixed ion-selective membranes or *vice versa*. Sodium ion-selective membranes were prioritized due to their established importance in electrode applications, with conclusions remaining unaffected by regardless of the ion type used (*e.g.*, K^+^, H^+^, Cl^−^). To construct working electrodes with membranes, an equal amount of ion-selective membrane was applied onto solid contacts. Further details regarding the electrode construction and cleaning processes, as well as information about various materials and membranes, can be found in ESI.†

The main device used in all experiments was an Autolab PGSTAT302N (Metrohm, Herisau, Switzerland). Cyclic voltammetry (CV), SPECS, and EIS were applied for systems with a potential window from −0.5 to 0.5 V (*versus* open circuit potential, *E*_ocp_). All original experimental data, including SPECS and 3D-electrochemical impedance spectroscopy, are listed in ESI.† All figures in the manuscript were calculated from the original electrochemical data presented in Fig. S1–S12.†

### 2.2 Theoretical section

In this study, fundamental electrochemical processes occurring at the electrode interface were deduced from electrochemical signals and described using mathematical equations. We divided these processes into two categories: (i) those dependent on electrode potential and (ii) those independent of electrode potential. The former on interfaces or in thin layers are influenced by changes in potential, such as redox reactions or intercalation, while the latter remain unaffected by variations in potential, including self-exchange reactions caused by redox couples, or physical diffusion. Despite possible interactions between different reactions, we can deconvolute electrochemical data (SPECS and EIS) to determine the contribution of each interfacial process and derive percentages quantitatively. In addition, this method depends on modelling electrochemical data using the induction method, which differs from traditional interface models that are based on physicochemical equations for deductive reasoning. Nevertheless, its fundamental assumptions can be listed:^28^

(1) The interfaces of sensors are all separate and homogeneous to guarantee that phenomena within the same interface are equivalent and those in different interfaces are continuous.

(2) Basic parameters, such as standard chemical potentials of chemical components, ionic mobilities, pressure, and temperature, are assumed to remain consistent across space and time.

(3) The ion flux is solely dependent on the gradient of ion concentration and that of the applied potential.

#### 2.2.1 Classifying data with machine learning

Given the large number of data sets that need to be fitted, it is essential to implement an algorithm for classifying data to reduce the workload. Principal component analysis (PCA) is used to reduce the dimensionality of the data sets, with phase angles, frequencies, and normalized capacitances being disregarded factors. Then a support vector machine (SVM) is utilized to measure how special the sample points within each classified dataset are, specifically by calculating the distances of support vectors. By identifying the maximum distance among all sample points, the most distinctive signal was selected to proceed to the next step.

All programs are compiled by Python (version 3.12), and the detailed results of classifying data sets are depicted in Fig. S13–S18 and Table S1.†

#### 2.2.2 DRT analysis

The experimental impedance of different types can be expressed as a function of relaxation times (*τ*), like eqn (1), and specific interfacial processes can be deconvoluted through analysis of distribution functions (*g*(*τ*)).^29,30^1
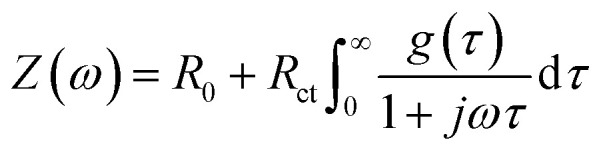
where *Z*(*ω*) is the real impedance data, *R*_0_ is the ohmic resistance on the solid–liquid interface, *R*_ct_ is the charge transfer resistance (*R*_0_ and *R*_ct_ may be denoted as different physical constants in various systems and require distinct methodologies for data processing), *τ* is the relaxation time constant, and *g*(*τ*) is the distribution function of *τ*.

Two preprocessing steps are necessary to ensure the accuracy of the data. First, due to the constraints imposed by regularization methods for dealing with eqn (1), the imaginary part of *Z*(*ω*) must approach zero at infinity. This implies that data representing diffusion, such as Warburg elements, must be subtracted.^31^ Afterwards, a test of the Kramers–Kronig relation should be conducted to confirm that the data conforms to the behaviour of linear time-invariant systems.^32^ To perform DRT analysis on processed data, DRTtools (open-source software developed by Wan *et al.*) is utilized to compute second Fredholm integral equations through radial basis function expansion.^33^

An example and analysis of the DRT simulation process are presented in Fig. S19 in ESI,† encompassing the deduction of impedance data and the analysis of DRT fitting results. Detailed results of the DRT analysis and interfacial modes are depicted in Tables S2 and S3.†

#### 2.2.3 SPECS analysis

By analysing SPECS, various types of current can be fitted during potential stepping processes. A simple representation of the SPECS model is provided in eqn (2):

2 *I*_T_ = *I*_C_ + *I*_D_ + *I*_R_

where *I*_T_ (unit: A) is the total current, *I*_C_ is the current of capacitive charging, *I*_D_ is the current of the diffusion process under different boundary conditions, *I*_R_ is the residual current. *I*_C_ and *I*_D_ can be represented as:^34^3
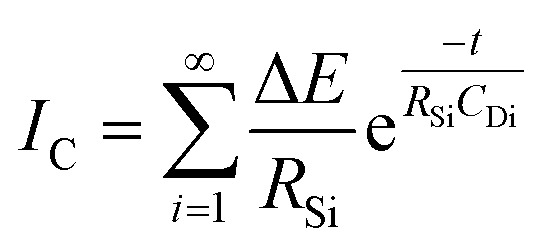
4
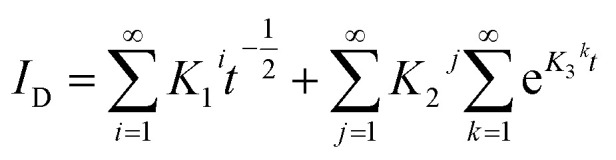
where Δ*E* (unit: V) is the applied potential (*versus E*_ocp_), *R*_Si_ (unit: Ω) and *C*_Di_ (unit: F) are the resistance and capacitance for capacitive interfacial processes, respectively, *t* (unit: s) is time, and *K*_1_, *K*_2_, and *K*_3_ are constants for diffusional interfacial processes. It should be emphasized that different summation symbols in eqn (3) and (4) have distinct meanings. The summation symbol in eqn (3) indicates the sum of some capacitive processes, and 
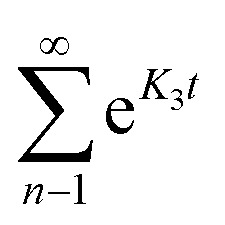
 in eqn (4) represents an infinite series under a single finite diffusion condition, with other summation symbols in eqn (4) indicating the sum of multiple interfacial processes. The global optimization procedure of this problem was resolved with OpenLu64 (open-source software).

## 3 Results and discussion

### 3.1 Comparisons between CVs and interfacial processes

**Fig. 1:**
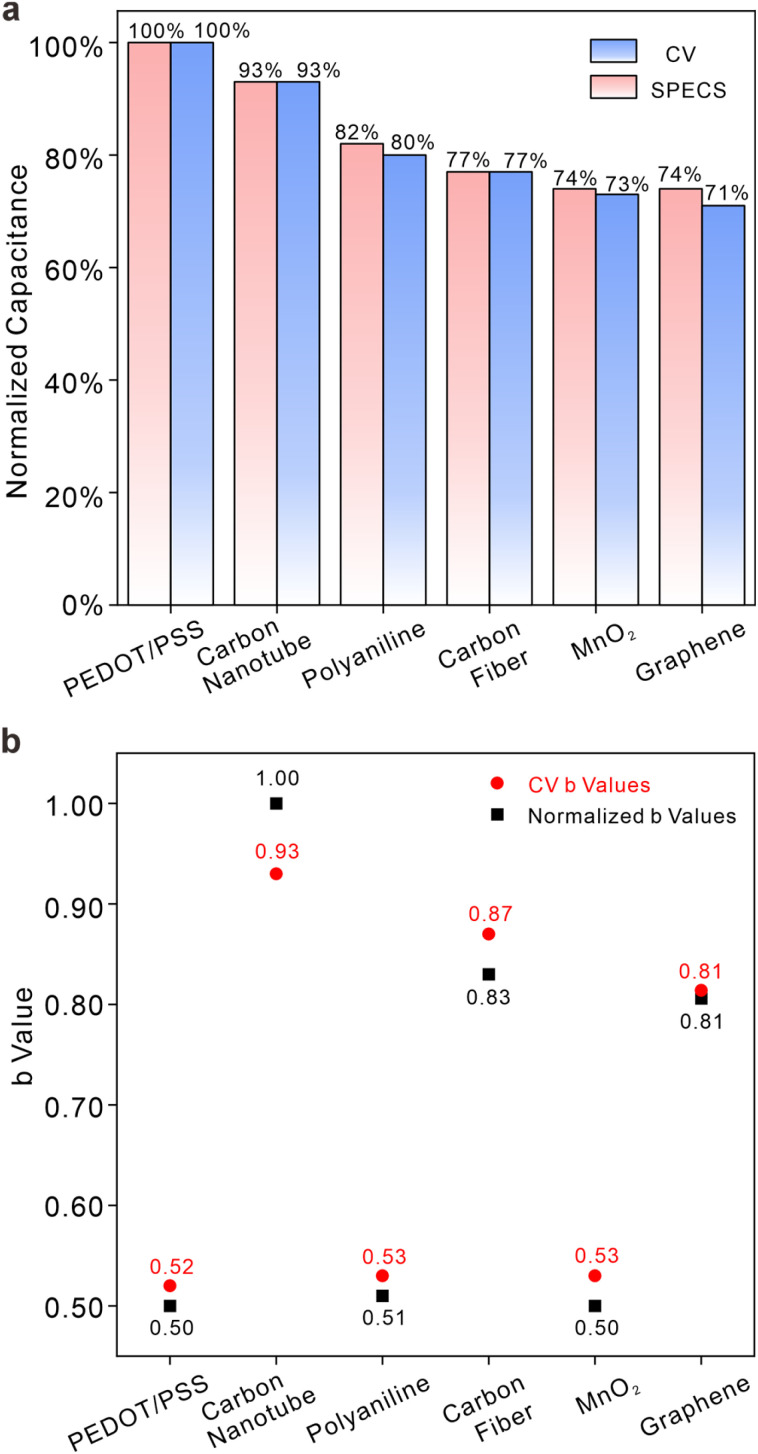
Comparisons between CVs and interfacial processes in membrane-less electrode systems. (a) A comparison of normalized charge between interfacial processes and CVs for six different materials in 0.1 M NaCl solutions. Blue bars represent the charge integrated from CVs, while red bars denote interface process analysis. (b) A comparison of *b*-values between interfacial processes and CVs for six different materials in 0.1 M NaCl solutions. Red cycles represent *b*-values derived from the correlation between scan rates and current peaks in CVs, while black squares indicate normalized *b*-values calculated based on the ratio of capacitive processes to redox processes.

Fig. 1a illustrates the consistent order of electric charge quantities at the electrode interface, in the absence of ion-selective membranes, as follows: PEDOT/PSS > carboxylated carbon nanotubes > polyaniline > carbon fiber > manganese dioxide (MnO_2_) > graphene. All capacitances for materials are calculated from integration of CV under a scan rate of 10 mV s^−1^ and interfacial process (*I*_C_ and *I*_D_) calculations in Fig. S20.† It is important to note that the integrated areas may vary with scan rates due to the transient characteristics of CV, with slower scan rates leading to better alignment between the two methods. Despite this, Fig. 1a suggests that both methods are equally capable of assessing charge storage capacity and giving similar results. Additionally, the calculated *b*-values in Fig. 1b further support the equivalence of these two methods in systems without membranes. In Fig. 1b, the normalized *b*-values drawn as black squares were calculated as the ratio of capacitive processes to redox processes and then normalized to a range of 0.5–1, indicating the property of capacitance at the interface (*i.e.*, as the value approaches 1, the dominance of the capacitive process over charge storage processes becomes more pronounced in comparison to the pseudocapacitive process; as the value approaches 0.5, the dominance of the pseudocapacitive process over charge storage processes becomes more pronounced in comparison to the capacitive process). For red cycles, the *b*-value indicates the relationship between peak current and scan rate (*i.e. I*_p_ = *av*^*b*^). A value of 1 suggests that interfacial processes are controlled by surface processes (*e.g.* capacitive processes), while a value of 0.5 suggests that interfacial processes are controlled by diffusion (*e.g.* pseudocapacitive processes). According to the traditional *b*-value method, carboxylated carbon nanotubes, carbon fiber and graphene belong to one category of material, while MnO_2_, polyaniline and PEDOT/PSS belong to another category. Although the normalized *b*-value method lacks a clear physical interpretation, it still categorizes the six samples into the same two groups as the traditional *b*-value method. As a result, both normalized *b*-values and traditional *b*-values depict the fundamental characteristics of electrode interfaces.

### 3.2 Analysis of SPECS results

It is evident that the two methods can be equally employed for analysing modified electrodes without membranes, and it will be demonstrated that interface process analysis, which is based on the deconvolution of complex interface phenomena, can be further utilized to quantitatively evaluate SCISE.

**Fig. 2:**
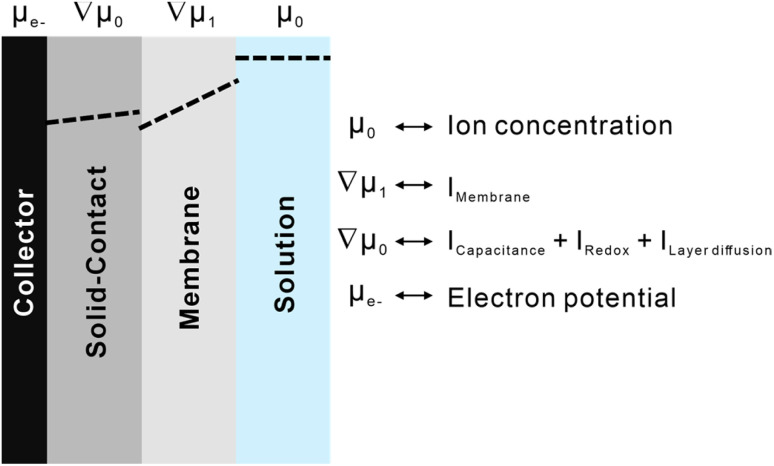
A schematic diagram describing the relationships between interfacial processes and simulated charging processes.

The analysed results of SPECS can be mathematically represented as a complex combination of functions related to various interface processes, still requiring physical explanations for widespread applicability. Fig. 2 describes a correlation between integral charges from SPECS and interface processes existing in the system of ion-selective electrodes. Chemical potentials of different components are represented by two constants and two vectors. Among them, the chemical potential of ions in the solution and that of electrons on the electrode are constant (*μ*_0_ and *μ*_e^−^_), while the chemical potentials of membranes and transductions are represented by gradient vectors (∇*μ*_1_ and ∇*μ*_2_). Each variable is continuous in its own phase and discrete on the interface. The chemical potentials and integrated charges associated with a specific part in electrochemical systems could be considered to be correlated. This indicates that the charging processes taking place at interfaces, and the change in chemical potentials resulting from ion concentrations represent the same interface phenomenon under different circumstances. As a result, capacitive processes, redox processes, and layer diffusion present in solid contacts collectively contribute to stabilizing potentials, which are influenced by changing chemical potentials. Additionally, membrane processes can be regarded as the diffusion of ions in a uniform medium. Although target ions were transported by ionophores, the influence on chemical potentials was mitigated by the solid contacts.

**Fig. 3:**
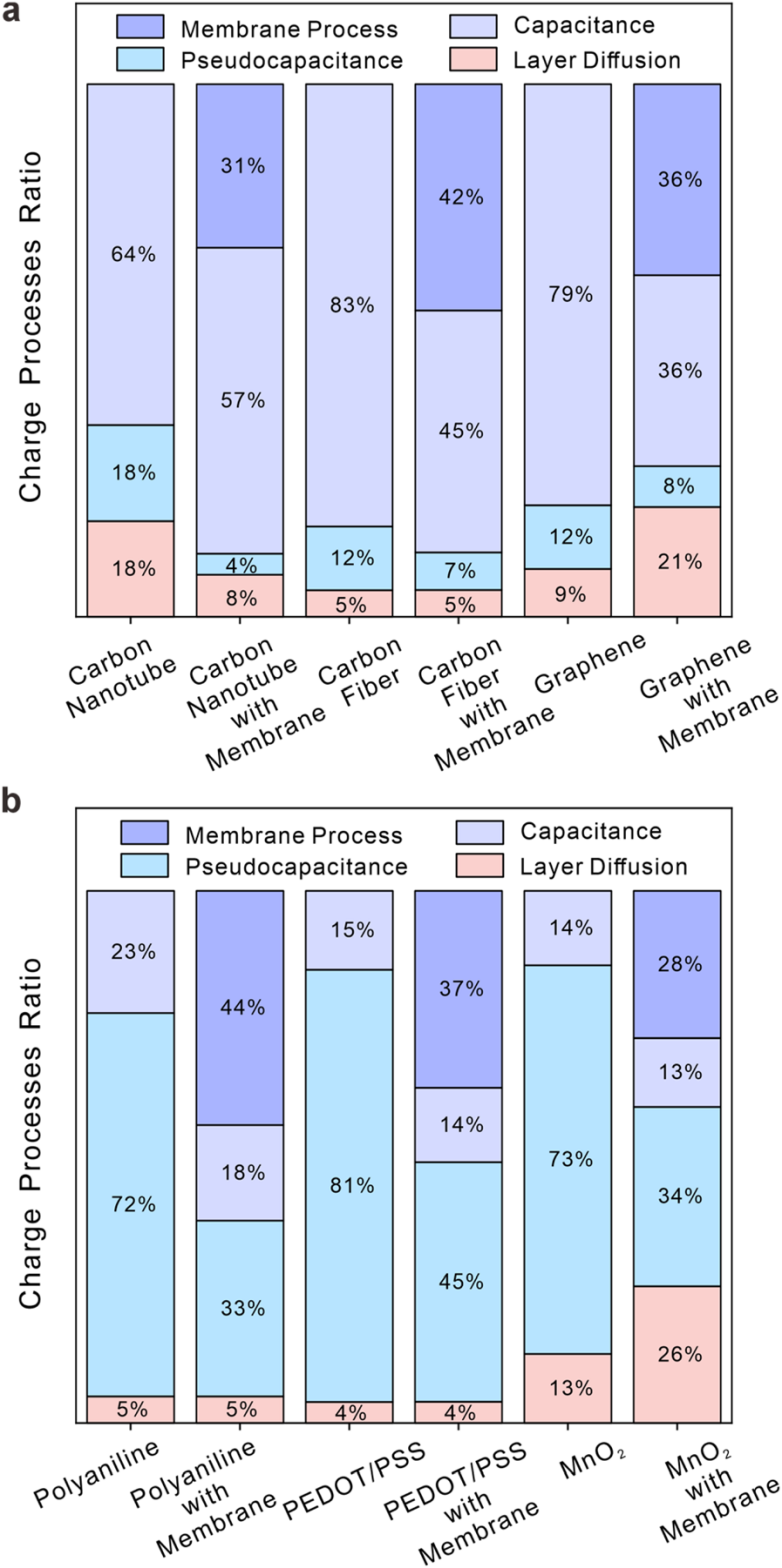
The ratio of different interface processes to total charging processes for different materials. Mauve bars represent capacitive processes, blue bars denote pseudocapacitive processes, red bars indicate processes for layer diffusion in materials, and purple bars correspond to membrane processes: (a) carboxylated carbon nanotubes, carbon fiber and graphene; (b) polyaniline, PEDOT/PSS, and MnO_2_. Both figures include the ratio of charge processes with and without membranes of each material.

After integrating processes with unique features or combining processes with comparable characteristics within these systems, the charge storage processes for six materials are illustrated in Fig. 3 (simulations for the individual material with and without membranes are listed in Fig. S21–S26†). Fig. 3a depicts the distribution of different charge storage processes within the overall charging processes for three carbon-based materials: carboxylated carbon nanotubes, carbon fiber, and graphene. Among them, capacitive processes are predominant in ion-selective systems, while pseudocapacitive processes and layer diffusion processes play a minor role. Fig. 3b illustrates distinct charging mechanisms of polyaniline, PEDOT/PSS, and MnO_2_. In contrast to carbon-based materials, pseudocapacitive processes are predominant factors in all charging processes of the three materials. It is evident that the energy storage mechanism of carbon-based materials exhibits significant differences from that of the other three materials. In other words, the former is attributed to capacitive processes, while the latter is driven by pseudocapacitive processes. The comparison of each material for the system with and without membranes in Fig. 3 reveals no significant alteration in the primary charging process of each material, suggesting that the addition of ion-selective membranes does not impact the energy storage mechanisms of solid contacts. The presence of membranes on electrodes, regardless of the material used, leads to limited ion fluxes and directions, ultimately reducing the overall charging process and causing significant membrane diffusion. It should be added that, as Fig. 3 shows, both graphene and MnO_2_ demonstrate substantial layer diffusion as a result of their unique layered structure at electrodes with membranes. Layer diffusion in solid materials is often considered a primary process for pseudo-capacitance but should be deemed insignificant in the context of ion detection studies without membranes due to its minimal impact. Conversely, it is crucial to take into account the layer diffusion of specific materials within ion-selective membranes, as it becomes apparent when all charging processes are attenuated. Although the inhibition of ion-selective membranes on the charging process has been obvious, how this limitation varies among different materials and what impact this difference has on the selection of solid contacts still needs to be studied.

### 3.3 Inhibitions of membranes for ion transduction

This section will demonstrate the impact of interfacial process symmetry on the properties of solid contacts with membranes and provide an inference for this influence, whether the material is “capacitive” or “pseudocapacitive”.

**Fig. 4:**
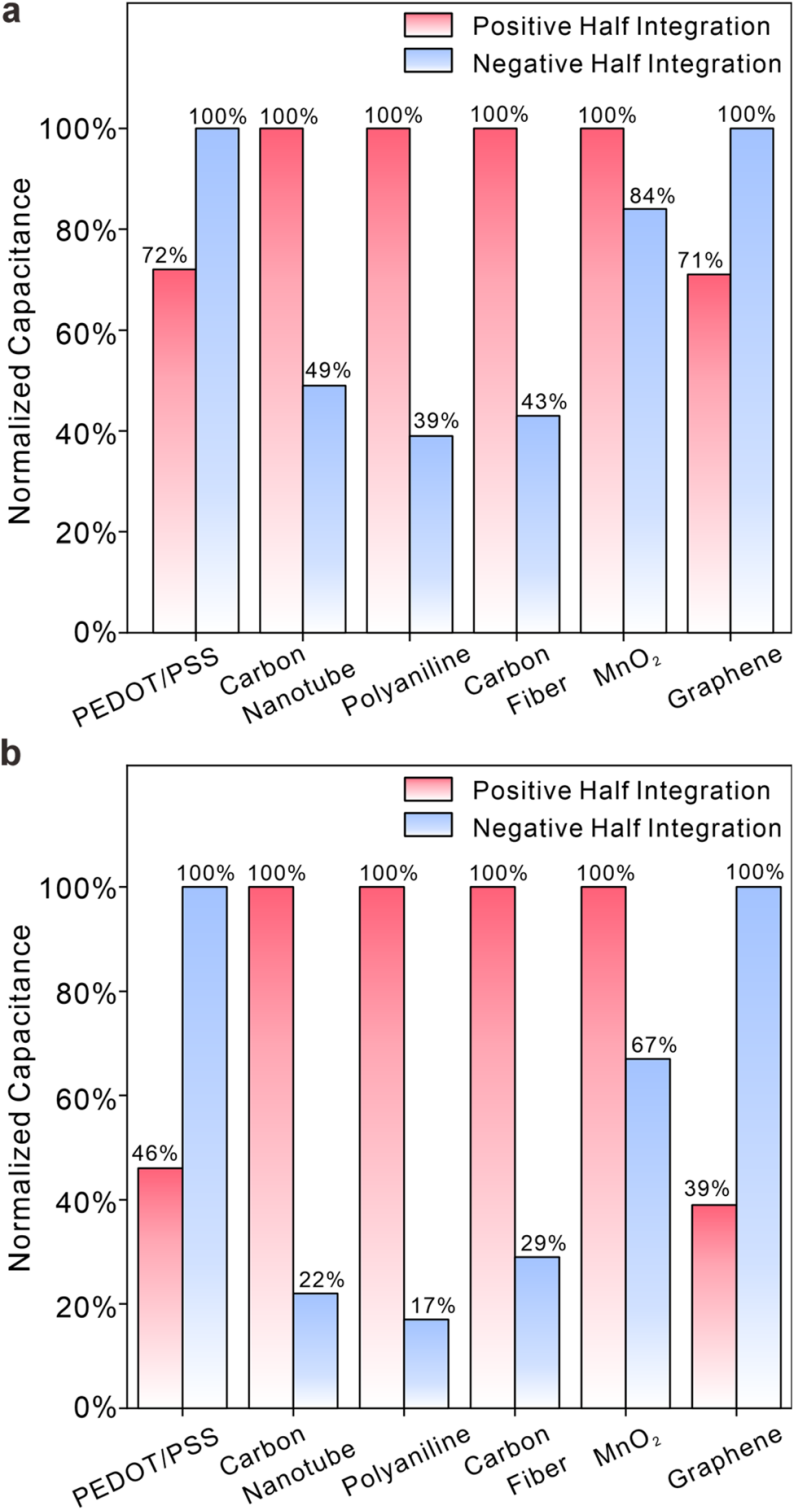
(a and b) A comparison of normalized charge between positive and negative overpotentials (*versus E*_ocp_) for six different materials with (a) and without (b) membranes in 0.1 M NaCl solutions. Blue bars represent the normalized amount of charge storage under negative potentials and red bars denote the normalized amount of charge storage under positive potentials.

Fig. 4a and b show the ratio of charges at electrodes modified with membranes under positive or negative overpotentials. Essentially, the data in Fig. 4a and b quantitatively represent the symmetry or asymmetry of transient processes at electrode interfaces, which can to some extent be interpreted as the relaxation characteristic of charge storage processes at electrode interfaces. In Fig. 4a, the normalized charge displays irregular fluctuations in relation to overpotential, depicted by red bars (half integration at positive overpotential) and blue bars (half integration at negative overpotential). Carboxylated carbon nanotubes, carbon fiber, and polyaniline exhibit significant disparities in symmetry. In contrast, PEDOT/PSS, graphene, and MnO_2_ demonstrate comparable charge integrals at both positive and negative potentials. When closely observed, the behaviour of graphene resembles that of a capacitor, indicating a physical effect independent of overpotential. MnO_2_ and PEDOT/PSS exhibit characteristics closer to surface redox reactions, and their symmetry can be attributed to the lack of need for additional ion diffusion in the solution. The asymmetry observed in carboxylated carbon nanotubes and carbon fiber stems from the modified functional groups, which can attract ions of opposite charge, resulting in an asymmetric charge storage process as the potential changes.^35^ The highly asymmetric charging process demonstrates the irreversible redox process that occurs in polyaniline, which is attributed to prolonged charging processes and the pH-dependent transition from the electrically conducting emeraldine salt form to the nonconducting emeraldine base form.^3^Fig. 4b presents the same phenomenon as Fig. 4a, except for the lack of membranes on the electrode. It is evident that the variation in normalized charge for different materials follows a consistent pattern but with more pronounced differences for each material. This seems to indicate that membranes give rise to the buffering effect, leading to a closer approach between capacitances at overpotentials of different signs.^36^

**Fig. 5:**
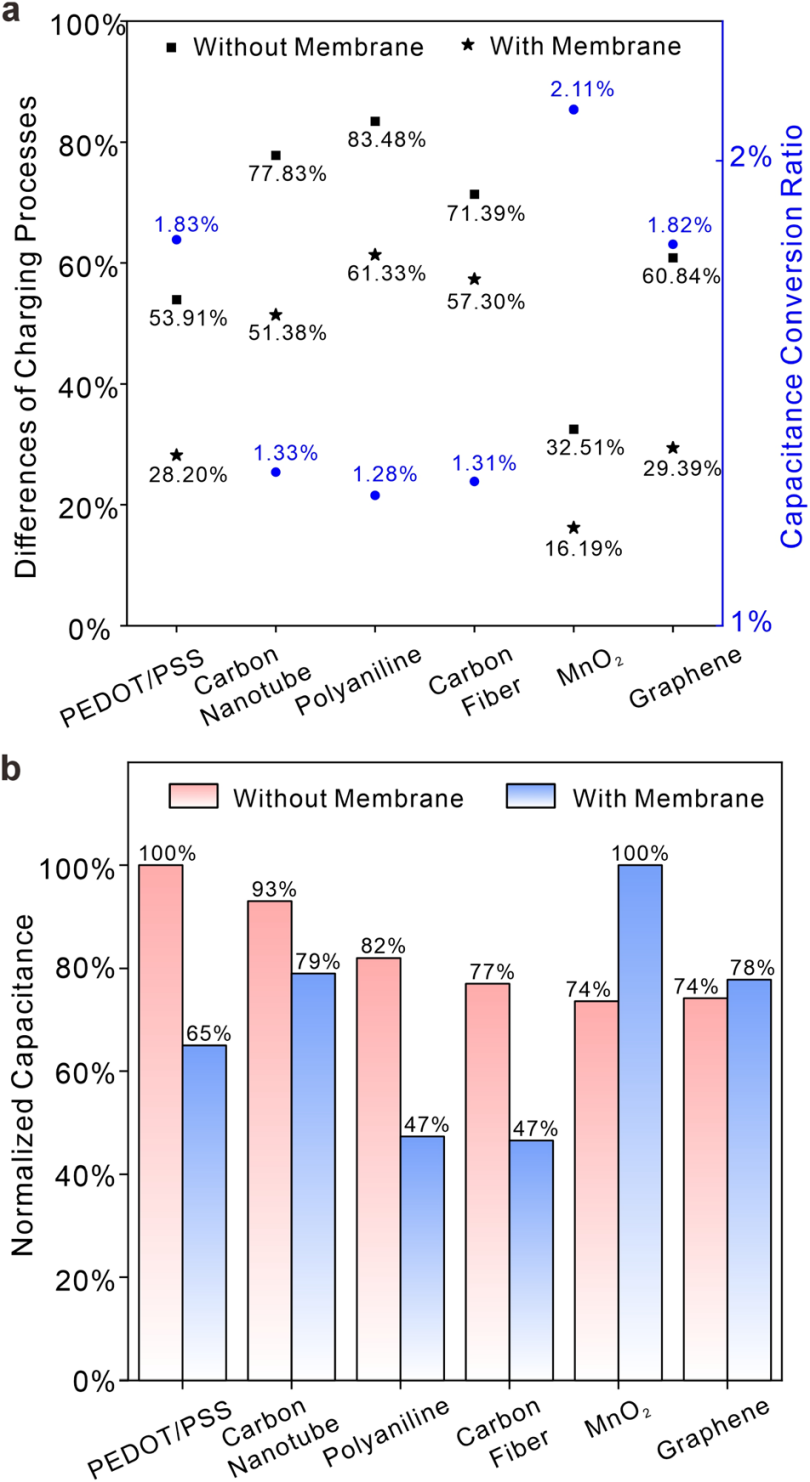
(a) Differences in capacitance processes at positive and negative overpotentials (left axis) and capacitive conversion ratios (right axis) for six different materials in 0.1 M NaCl solutions. Black squares (without membranes) and stars (with membranes) indicate magnitudes of variations in charge storage capacity between positive and negative potentials, while blue circles represent ratios in capacitances with and without a membrane. (b) A comparison of normalized charge between electrodes with and without membranes for six different materials in 0.1 M NaCl solutions. Blue and red bars represent the normalized amount of charge storage with the membrane or absence the membrane, respectively.

**Fig. 6:**
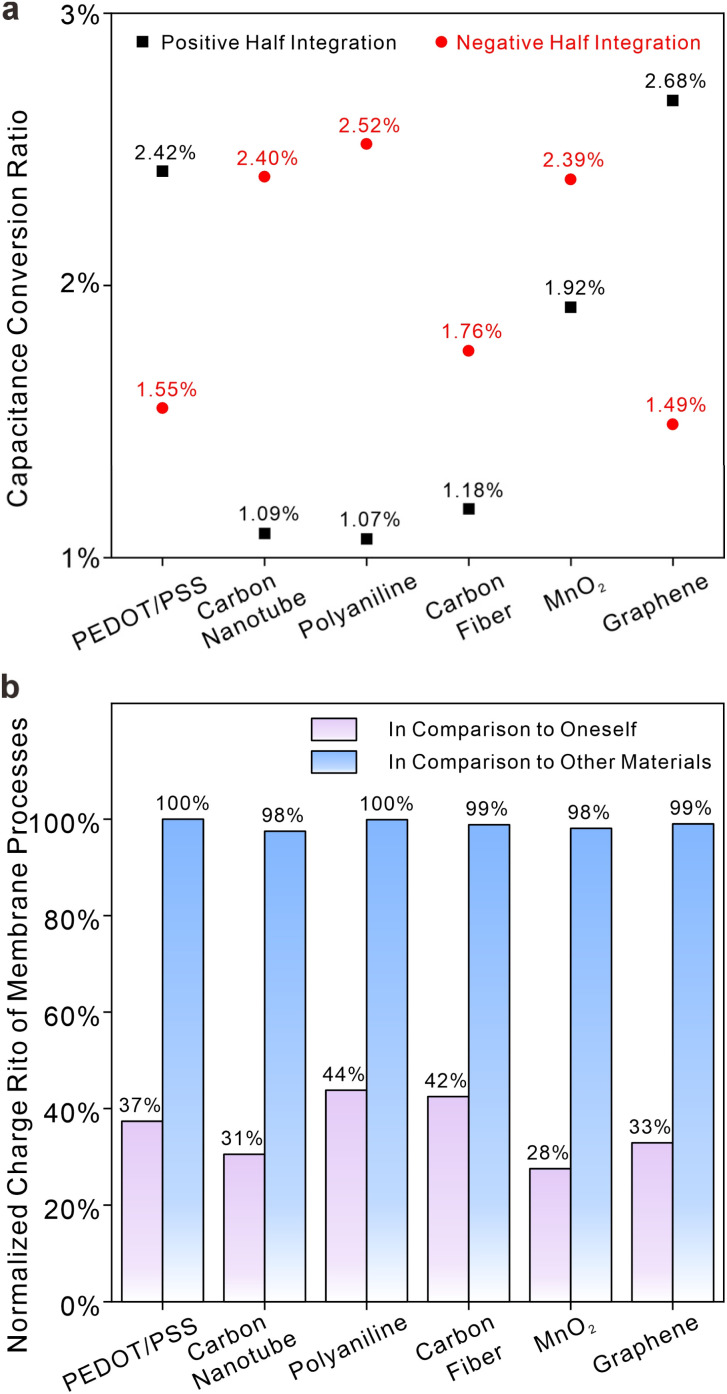
(a) Capacitive conversion ratios under positive (black squares) and negative (red cycles) overpotentials for six different materials in 0.1 M NaCl solutions. (b) Ratios of membrane processes in total charge storage processes in comparison to oneself (purple bars) and normalized charge of membrane processes in comparison to other materials (blue bars).

Fig. 5a defines two important parameters: the difference in capacitance processes at different overpotentials (DCPDO) and capacitive conversion ratio (CCR). The former is determined by the normalized difference in charge storage under overpotentials of different signs, while the latter is calculated based on the quotient of charge storage quantities of electrode systems with or without membranes. Black squares (without membranes) and stars (with membranes) indicate the magnitudes of variations in charge storage capacity between positive and negative potentials, while blue circles represent ratios in capacitances with and without a membrane. Although the CCRs of various materials are generally low due to the presence of membranes, they can still be categorized into two distinct types, each with its own DCPDO. Larger a DCPDOs are often associated with smaller CCRs. PEDOT/PSS, graphene and MnO_2_ exhibit lower DCPDO values and higher capacitance conversion ratios, both with and without film. Carbon fiber, carboxylated carbon nanotubes and polyaniline do the opposite. In other words, solid contacts materials that have good symmetry with respect to positive and negative half overpotential integrals are prone to exhibit larger capacitance or pseudo-capacitance for potential stabilization in real systems involving membranes. Fig. 5b illustrates the normalized charge storage of six different material systems with and without membranes, showing significant variations in the sequence of charge storage. The sequence for systems without membranes is as follows: PEDOT/PSS > carboxylated carbon nanotubes > polyaniline > carbon fiber > MnO_2_ > graphene. The sequence for systems with membranes is as follows: MnO_2_ > carboxylated carbon nanotubes > graphene > PEDOT/PSS > carbon fiber > polyaniline. This means that the selection of material for the solid contacts may be erroneous if it follows the sequence of charge storage ability of modified electrodes (*i.e.*, without membranes). It should be noted that the capacitance sequence in Fig. 5b represents the total capacitance encompassing all interface processes, while the capacitance conversion ratio in Fig. 5a refers to the conversion rate of the main capacitance process. Thus, the magnitude of the total capacitance in the membrane does not solely depend on the conversion rate of the capacitance process. Nevertheless, when the main charging process constitutes a large proportion of total capacitance processes, the capacitance conversion ratio can give a good evaluation of the capacitance in membranes. In Fig. 6a, CCRs for positive and negative overpotentials were calculated. It is apparent that a higher normalized charge value corresponds to a lower conversion ratio for the same material. This suggests that ion-selective membranes exert a more pronounced inhibitory effect on processes involving relatively high charge storage. The symmetry of the integral charge at potentials of different signs can be attributed to the distinct properties of the material itself at the thermodynamic scale under different overpotentials, while the disparity between membrane and non-membrane systems is a result of kinetic limitations on ion transport imposed by the ion-selective membrane, leading to slow charge storage on the solid contacts and a reduction in symmetry differences. Furthermore, Fig. 6b illustrates the normalized proportion of each material's membrane process in its own charging process and the normalized proportion of the membrane process prior to the different materials. It is obvious that membrane processes in different materials are almost quantitatively equivalent, although membrane processes account for quite different proportions in the total charge processes of each material. This appears to offer a method for evaluating the overall capacitance. Under the same circumstances, the interface with a lower ratio of membrane processes has greater overall capacitance. Given the similar quantities of charge in membrane processes observed across various materials and considering that a certain proportion of the membrane charge process occurs simultaneously at different solid contacts, it can be deduced that the ion-selective membrane serves as a current-limiting device toward ion fluxes. This inference can account for the variability in the magnitude sequence of charge storage processes in electrodes with or without membranes, as depicted in Fig. 5b. Meanwhile, this phenomenon may also suggest a maximum capacitance limit for solid contacts, as membrane processes exhibit relative stability, and capacitive processes are unable to surpass this limit in terms of kinetic aspects.

## 4 Conclusions

In this study, limitations in the capacitance conversion ratios of materials were observed through an integrated algorithm, which revealed that ion-selective membranes reduce the capacitance of solid contacts in SCISE systems. The EIS data were classified into multiple species using machine learning cluster methods, followed by DRT analysis to develop models required for SPECS simulations. After quantifying the charge storage processes, it becomes feasible to evaluate the performance of various types of solid contact. It was observed that the symmetry in charge storage processes among different materials can indicate their capacitance conversion ratio in systems with or without membranes. This is because ion-selective membranes have an uneven impact on charge storage processes in different materials under varying overpotentials. As capacitance increases, the membrane becomes more restrictive, particularly affecting materials with asymmetrical storage properties. In essence, the ion-selective membranes serve as a limiting factor within the overall interface system, as solid contacts need to process material and charge exchange from the bulk solution. Therefore, in addition to hydrophobicity and large capacitance, the development of solid contacts should prioritize materials that meet the following criteria:

(1) Charge storage processes should remain relatively balanced at overpotentials of different signs.

(2) The charge storage mechanism should consider the extent to which the membrane will restrict the charging process.

Additionally, the integrated algorithm utilized in this study is based on electrode interface kinetics, indicating that this approach may also serve as a widely applicable strategy across various electrochemical domains. This work, which combines experimental data analysis, numerical simulation, and machine learning, enhances the comprehension of dynamic processes at electrode interfaces, and ultimately strengthens the connection between analytical chemistry and physical chemistry.

## Author contributions

Rui-Ze Xia: conceptualization, investigation, validation, data curation, writing – original draft. Xin Cai: electrodes preparation, validation, writing – review & editing. Jing-Yi Lin: writing – review & editing. Yong-Huan Zhao: writing – review & editing. Zi-Hao Liu: writing – review & editing. Chen-Lu Wang: writing – review & editing. Shi-Hua Chen: supervision, funding acquisition, resources. Meng Yang: supervision, funding acquisition, resources. Zong-Yin Song: supervision, funding acquisition, resources. Pei-Hua Li: supervision, funding acquisition, resources, writing – review & editing. Xing-Jiu Huang: supervision, funding acquisition, resources, writing – review & editing.

## Conflicts of interest

There are no conflicts to declare.

## Supplementary Material

SC-016-D5SC01241D-s001

## Data Availability

The code for DRTtools can be found at https://github.com/ciuccislab/DRTtools with https://doi.org/10.1016/j.electacta.2015.09.097. The version of the code employed for this study is version V8. The code for OpenLu64 can be found at http://www.forcal.net/sysm/lu2/openlu.htm with http://www.forcal.net/sysm/lu2/oluhtm/user_openlu.htm. The version of the code employed for this study is version V2.0. The original data supporting this article have been included as part of the ESI.†
