# Supplementary material for: Inhibitions imposed by kinetic constraints of membranes in all-solid-state ion-selective electrodes: characteristics of interfacial capacitance in solid contacts
Source: Chem Sci. 2025 Apr 30;16(23):10245–54. doi: 10.1039/d5sc01241d (PMC12067092; doi:10.1039/d5sc01241d)
Supplement: SC-016-D5SC01241D-s001 [file SC-016-D5SC01241D-s001.pdf]

Supplementary Information (SI) for Chemical Science.
This journal is © The Royal Society of Chemistry 2025

Supporting information for

Inhibitions imposed by kinetic constraints of membranes in all-solid-state ion-selective electrodes: characteristics of interfacial capacitances in solid contacts

Rui-Ze Xia,[§ab] Xin Cai,[§ab] Jing-Yi Lin,[a] Yong-Huan Zhao,[ab] Zi-Hao Liu,[ab] Chen-Lu Wang,[ab] Shi-Hua Chen,[*a] Meng Yang,[*a] Zong-Yin Song,[*a] Pei-Hua Li,[*a] and Xing-Jiu Huang,[*ab]

[a] Key Laboratory of Environmental Optics and Technology, And Environmental Materials and Pollution Control Laboratory, Institute of Solid State Physics, HFIPS, Chinese Academy of Sciences, Hefei 230031, China

[b] Department of Materials Science and Engineering, University of Science and Technology of China, Hefei, 230026, China

[*] Corresponding author

[§] Rui-Ze Xia and Xin Cai contributed equally to this work.

E-mail: chenshh@issp.ac.cn (Shi-Hua Chen), myang@iim.ac.cn (Meng Yang), zysong@issp.ac.cn (Zong-Yin Song), peihuali@issp.ac.cn (Pei-Hua Li), xingjiuhuang@iim.ac.cn (Xing-Jiu Huang)

Tel.: +86 551 6559 1167; Fax: +86 551 6559 2420.

Contents

1. Figures

Fig. S1-S6 Step potential electrochemical spectroscopies and 3D-electrochemical impedance spectroscopies for different materials.

Fig. S7-S12 Impedance of phase angles and normalized capacitances for different materials.

Fig. S13-S18 Classifying data sets through machine learning.

Fig. S19 The deduction of impedance data and the analysis of DRT fitting results.

Fig. S20 Cyclic voltammetry (CVs) and interfacial processes in membrane-less electrode systems.

Fig. S21-S26 Results of SPECSs for materials with symmetric primary charging processes.

2. Tables

Table S1 Table of classification

Table S2 Table of interfacial models

Table S3 Table of interfacial parameters

3. Experimental section

1. Figures

1.1 Fig. S1-S6

Step potential electrochemical spectroscopies and 3D-electrochemical impedance spectroscopies for different materials. Fig. S1 is carboxylated carbon nanotube, Fig. S2 is carbon fiber, Fig. S3 is graphene, Fig. S4 is polyaniline, Fig. S5 is MnO_2 and Fig. S6 is PEDOT/PSS.

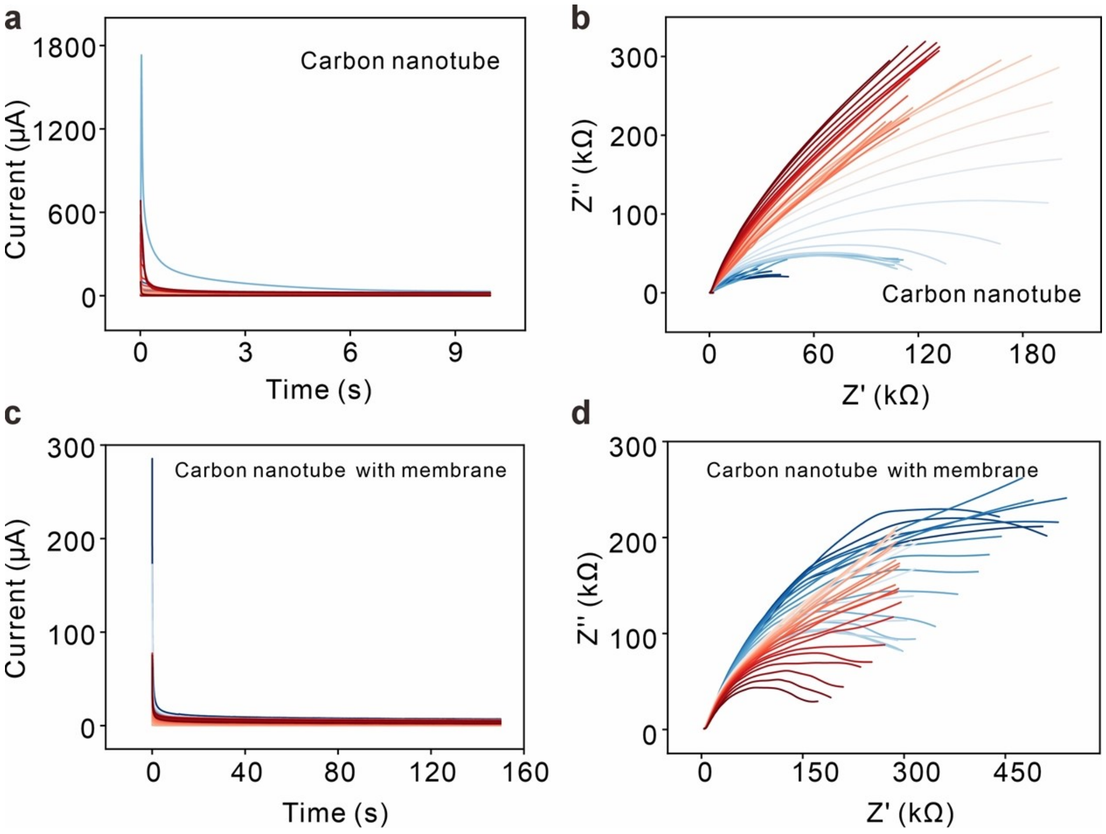

Fig. S1 (a) Step potential electrochemical spectroscopy for carboxylated carbon nanotube without membranes. (b) 3D-electrochemical impedance spectroscopy for carboxylated carbon nanotube without membranes. (c) Step potential electrochemical spectroscopy for carboxylated carbon nanotube with membranes. (d) 3D-electrochemical impedance spectroscopy for carboxylated carbon nanotube with membranes.

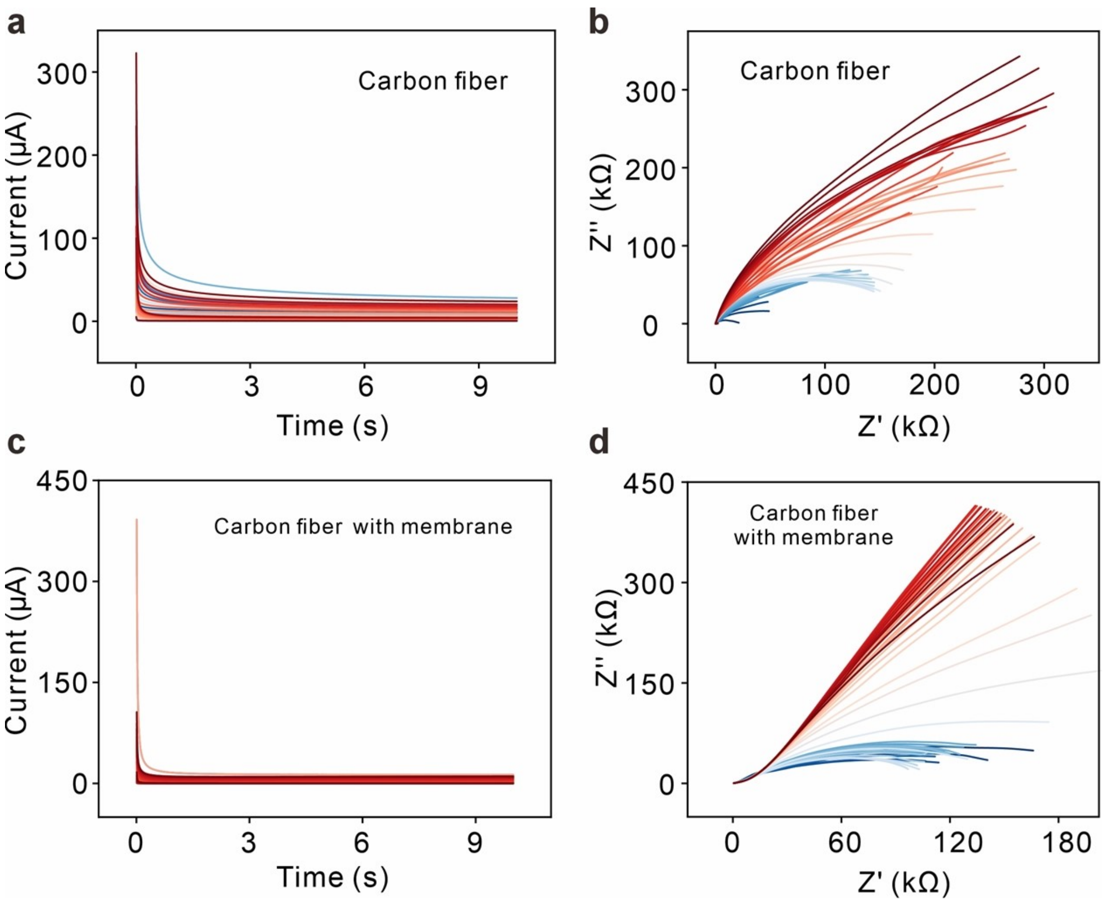

Fig. S2 (a) Step potential electrochemical spectroscopy for carbon fiber without membranes. (b) 3D-electrochemical impedance spectroscopy for carbon fiber without membranes. (c) Step potential electrochemical spectroscopy for carbon fiber with membranes. (d) 3D-electrochemical impedance spectroscopy for carbon fiber with membranes.

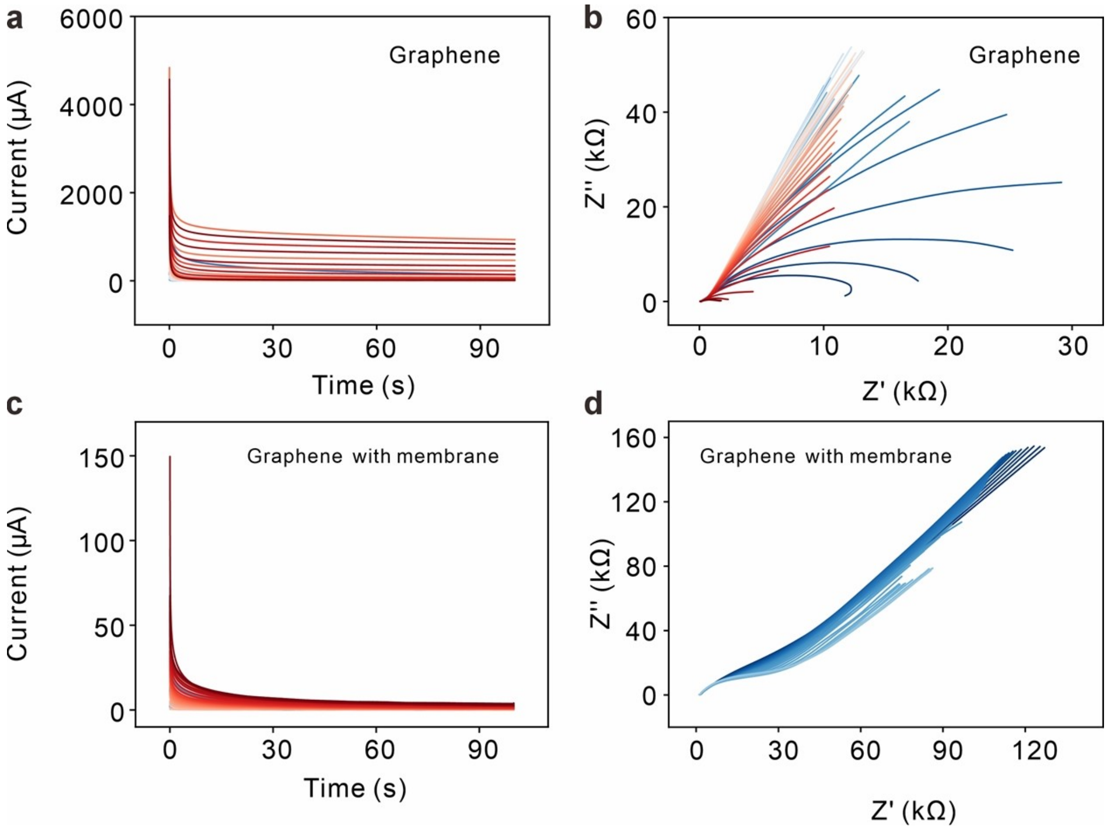

Fig. S3 (a) Step potential electrochemical spectroscopy for graphene without membranes. (b) 3D-electrochemical impedance spectroscopy for graphene without membranes. (c) Step potential electrochemical spectroscopy for graphene with membranes. (d) 3D-electrochemical impedance spectroscopy for graphene with membranes.

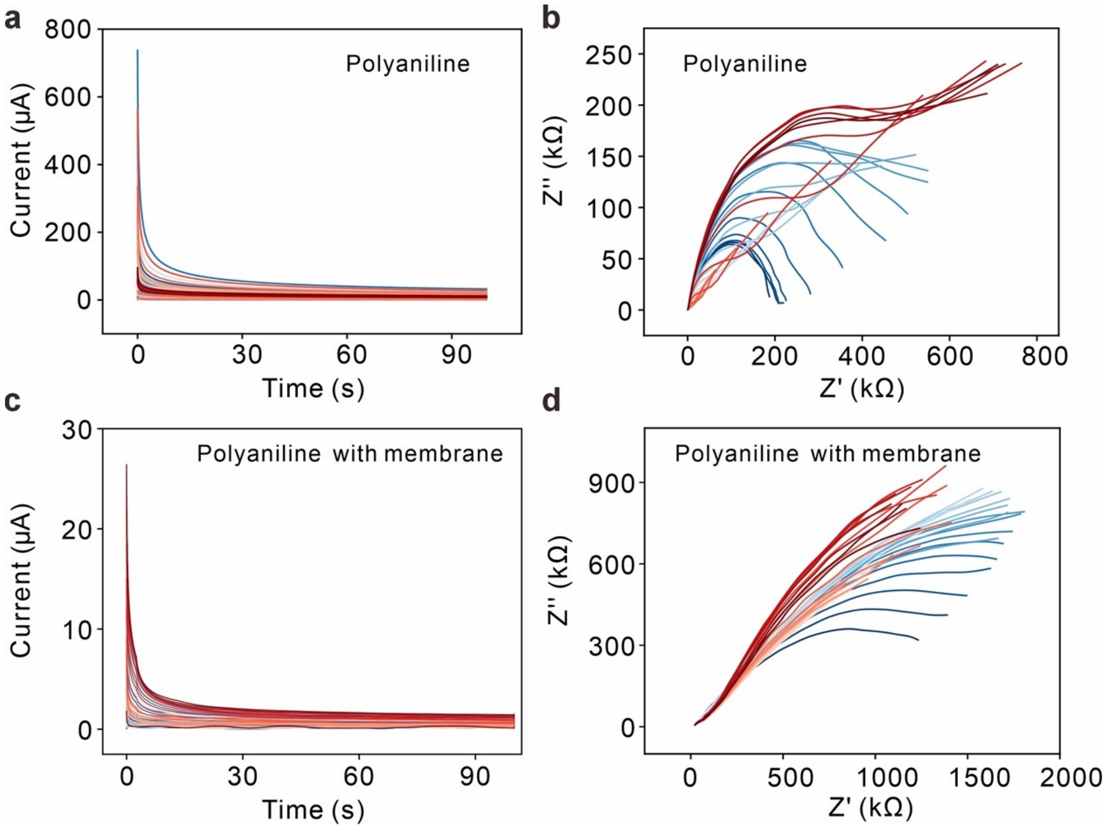

Fig. S4 (a) Step potential electrochemical spectroscopy for polyaniline without membranes. (b) 3D-electrochemical impedance spectroscopy for polyaniline without membranes. (c) Step potential electrochemical spectroscopy for polyaniline with membranes. (d) 3D-electrochemical impedance spectroscopy for polyaniline with membranes.

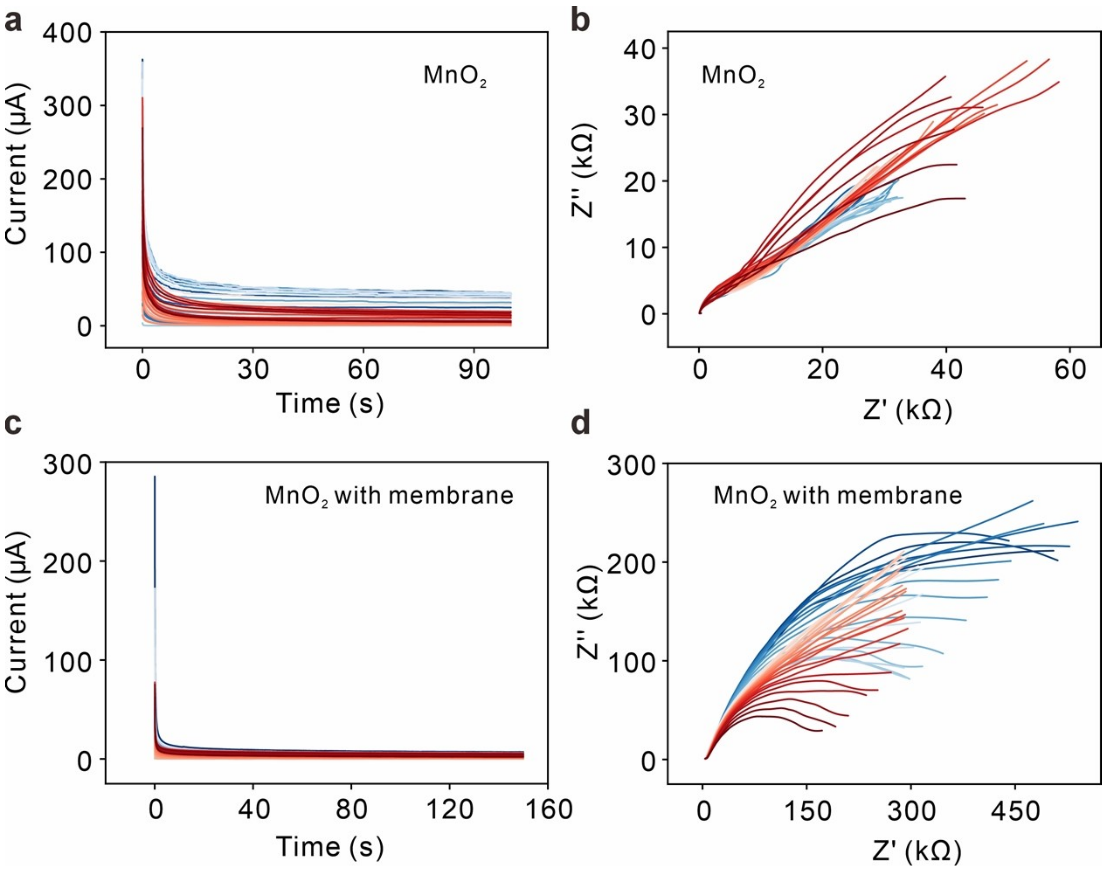

Fig. S5 (a) Step potential electrochemical spectroscopy for MnO_2 without membranes. (b) 3D-electrochemical impedance spectroscopy for MnO_2 without membranes. (c) Step potential electrochemical spectroscopy for MnO_2 with membranes. (d) 3D-electrochemical impedance spectroscopy for MnO_2 with membranes.

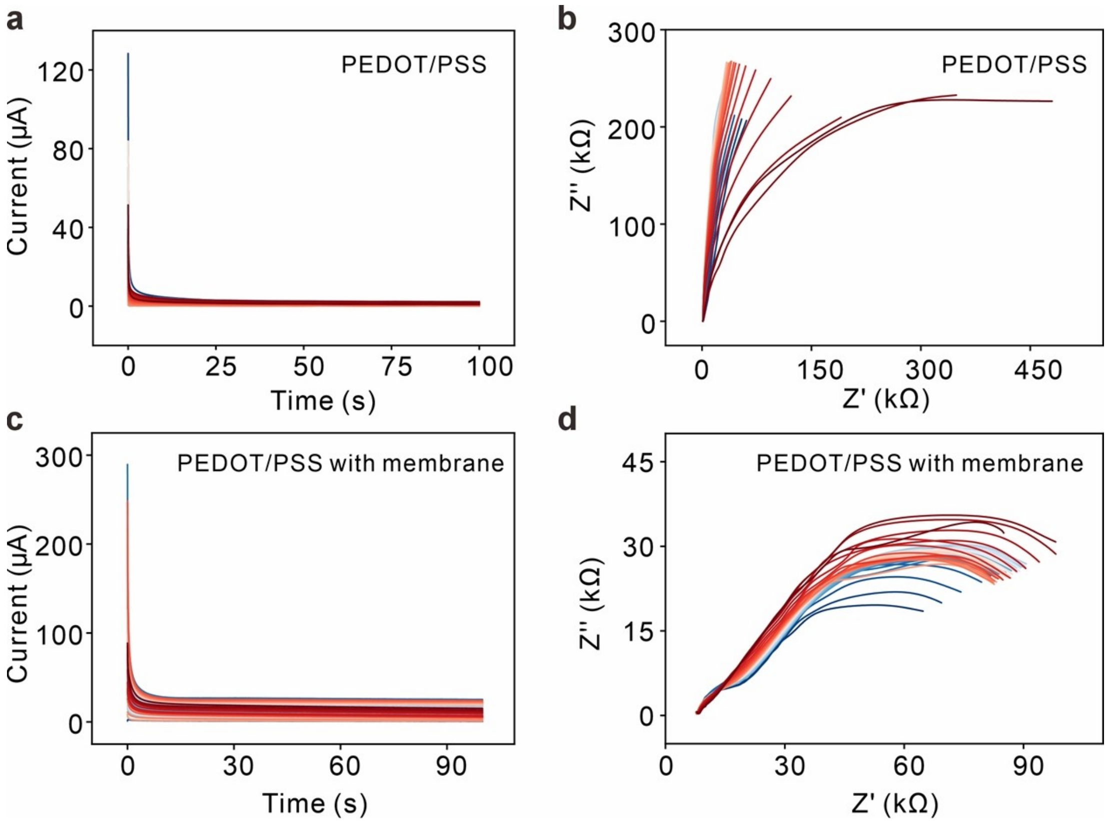

Fig. S6 (a) Step potential electrochemical spectroscopy for PEDOT/PSS without membranes. (b) 3D-electrochemical impedance spectroscopy for PEDOT/PSS without membranes. (c) Step potential electrochemical spectroscopy for PEDOT/PSS with membranes. (d) 3D-electrochemical impedance spectroscopy for PEDOT/PSS with membranes.

1.2 Fig. S7-S12

Impedance of phase angles and normalized capacitances for different materials. Fig. S7 is carboxylated carbon nanotube, Fig. S8 is carbon fiber, Fig. S9 is graphene, Fig. S10 is polyaniline, Fig. S11 is MnO_2 and Fig. S12 is PEDOT/PSS.

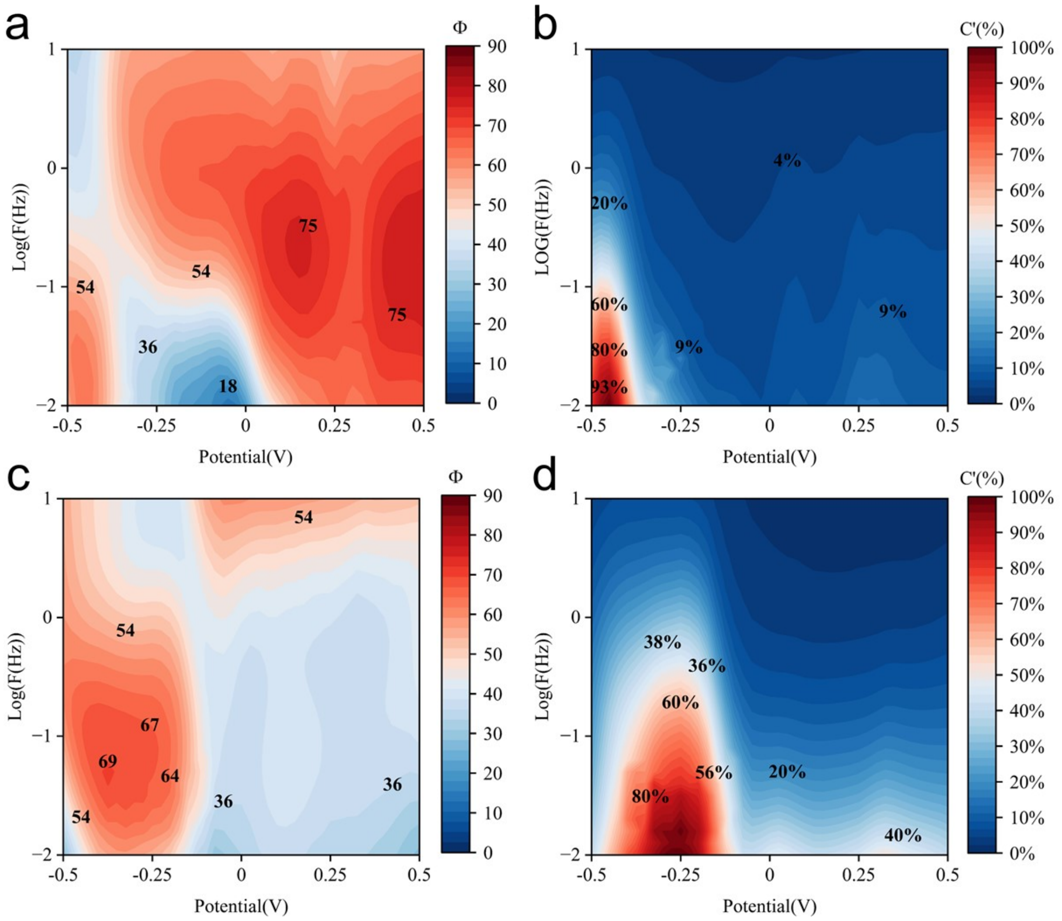

Fig. S7 (a) Phase angles for carboxylated carbon nanotube without membranes. (b) Normalized capacitance for carboxylated carbon nanotube without membranes. (c) Phase angles for carboxylated carbon nanotube with membranes. (d) Normalized capacitance for carboxylated carbon nanotube with membranes.

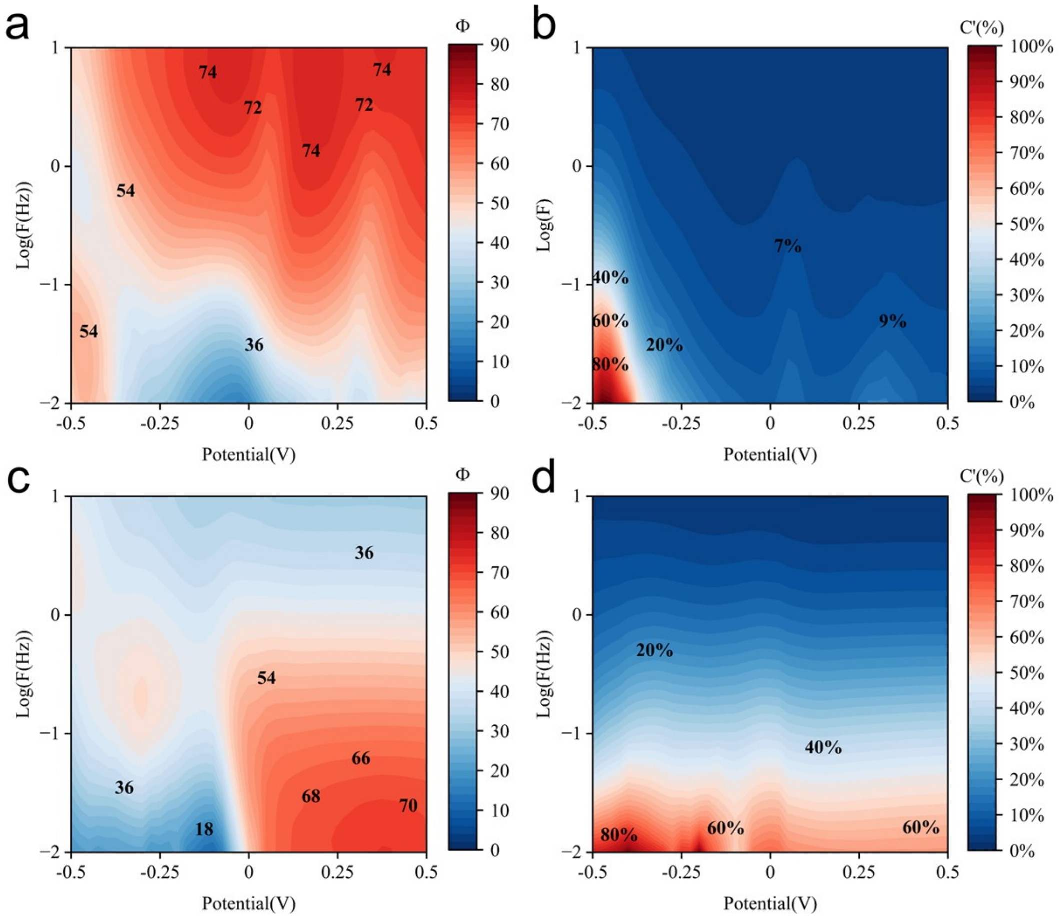

Fig. S8 (a) Phase angles for carbon fiber without membranes. (b) Normalized capacitance for carbon fiber without membranes. (c) Phase angles for carbon fiber with membranes. (d) Normalized capacitance for carbon fiber with membranes.

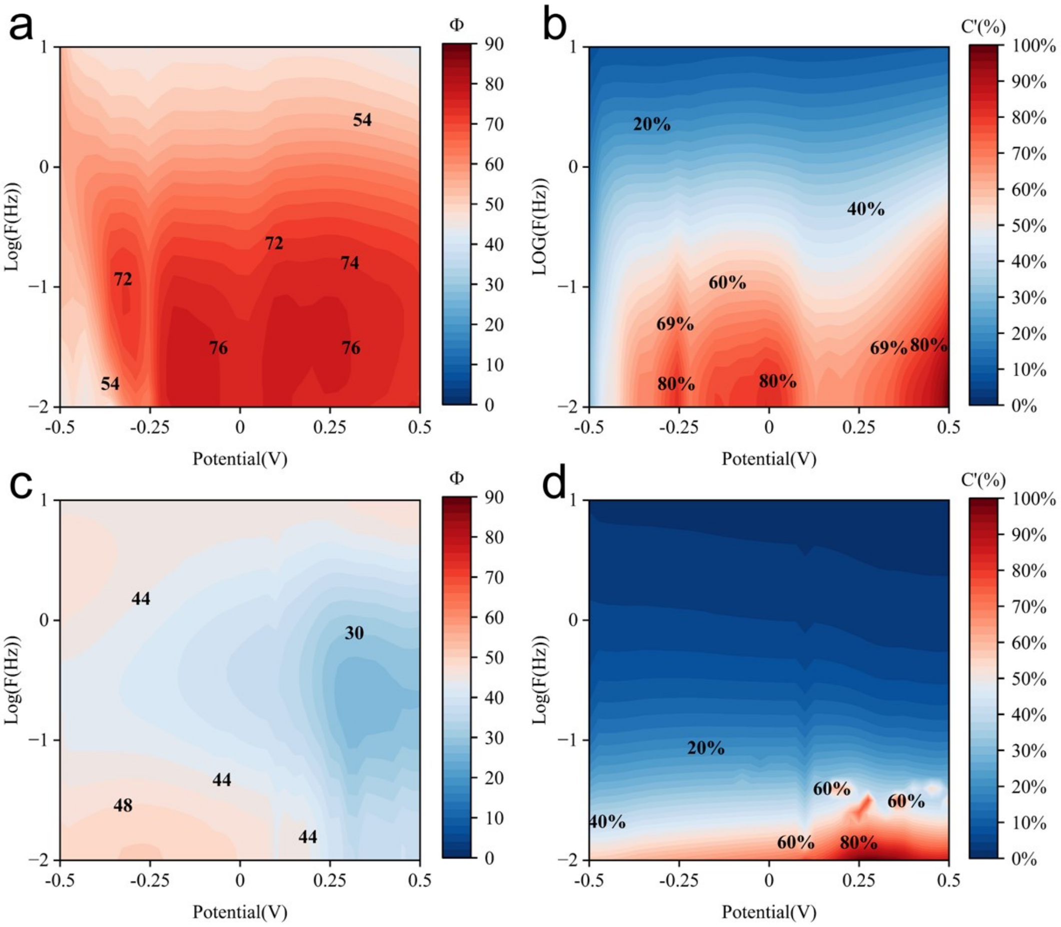

Fig. S9 (a) Phase angles for graphene without membranes. (b) Normalized capacitance for graphene without membranes. (c) Phase angles for graphene with membranes. (d) Normalized capacitance for graphene with membranes.

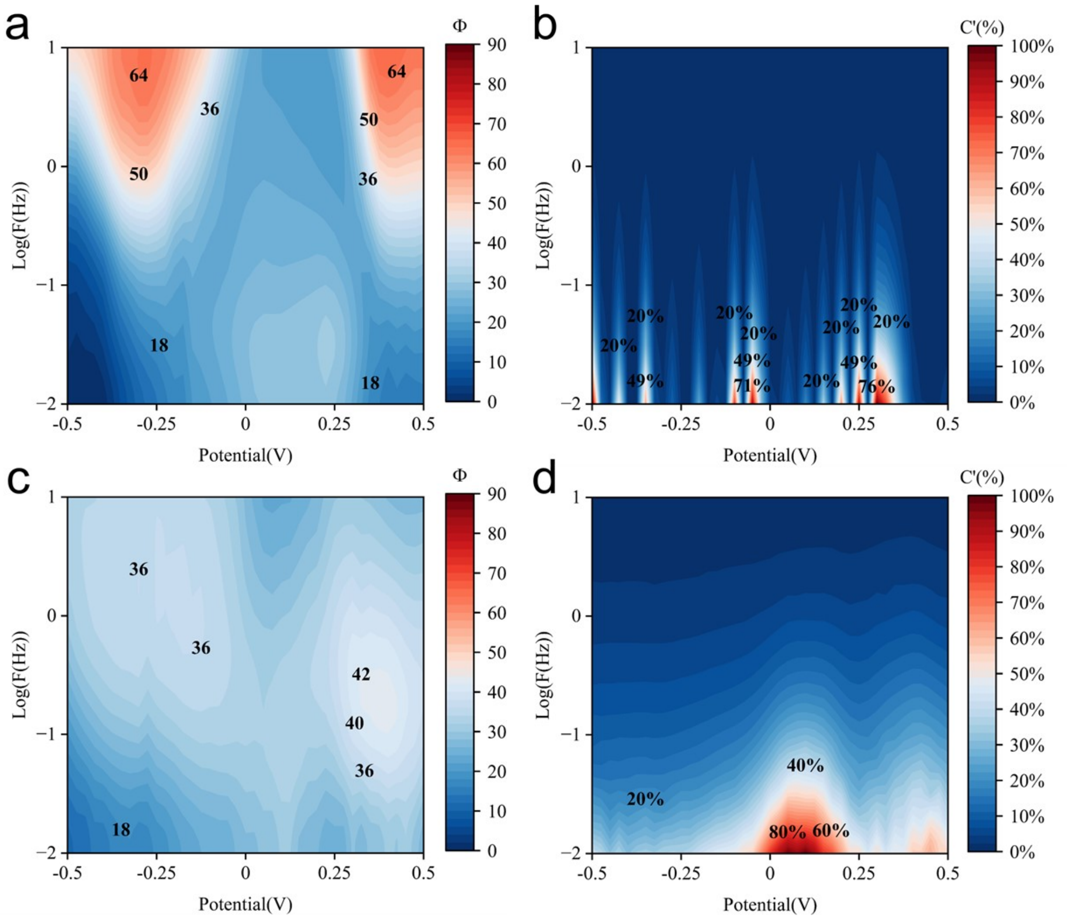

Fig. S10 (a) Phase angles for polyaniline without membranes. (b) Normalized capacitance for polyaniline without membranes. (c) Phase angles for polyaniline with membranes. (d) Normalized capacitance for polyaniline with membranes.

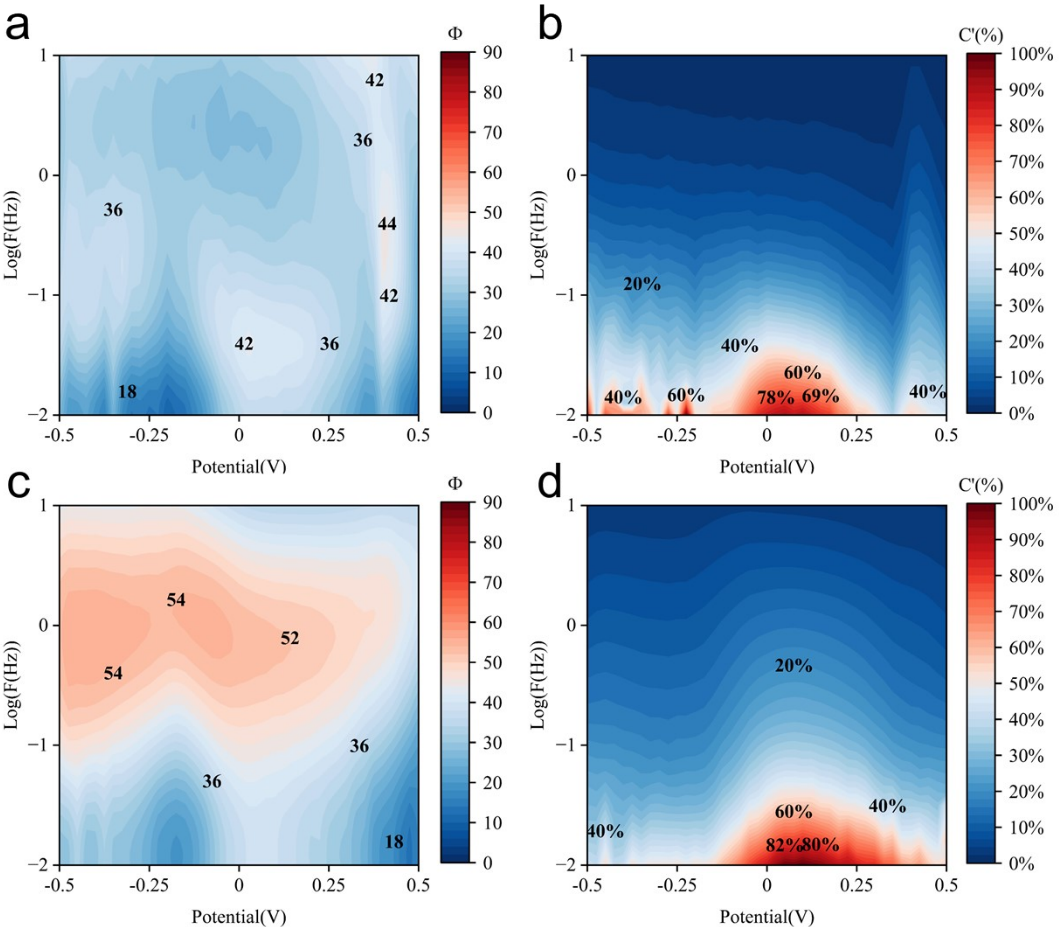

Fig. S11 (a) Phase angles for MnO_2 without membranes. (b) Normalized capacitance for MnO_2 without membranes. (c) Phase angles for MnO_2 with membranes. (d) Normalized capacitance for MnO_2 with membranes.

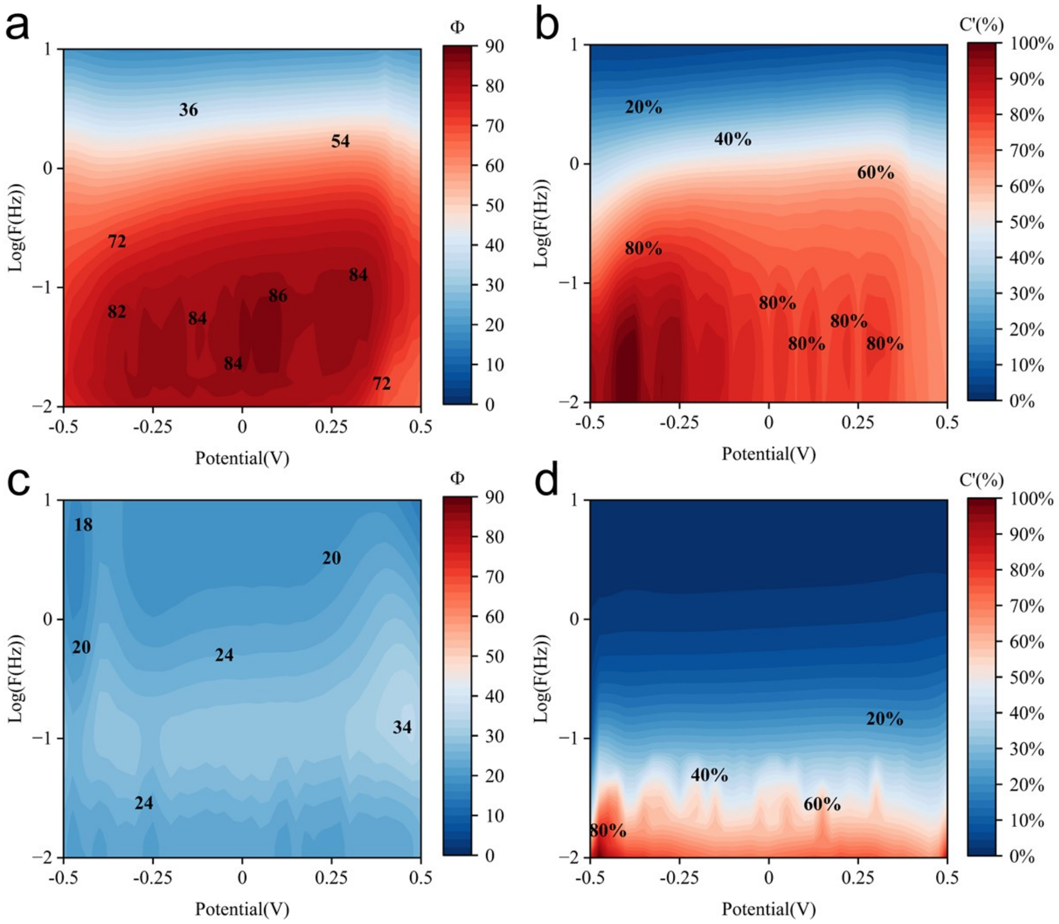

Fig. S12 (a) Phase angles for PEDOT/PSS without membranes. (b) Normalized capacitance for PEDOT/PSS without membranes. (c) Phase angles for PEDOT/PSS with membranes. (d) Normalized capacitance for PEDOT/PSS with membranes.

1.3 Fig. S13-S18

Figures below are data sets classified through machine learning, and different symbols represent different types. Specific classification status should be referred to Table S1. Fig. S13 is carboxylated carbon nanotube, Fig. S14 is carbon fiber, Fig. S15 is graphene, Fig. S16 is polyaniline, Fig. S17 is MnO_2, and Fig. S18 is PEDOT/PSS.

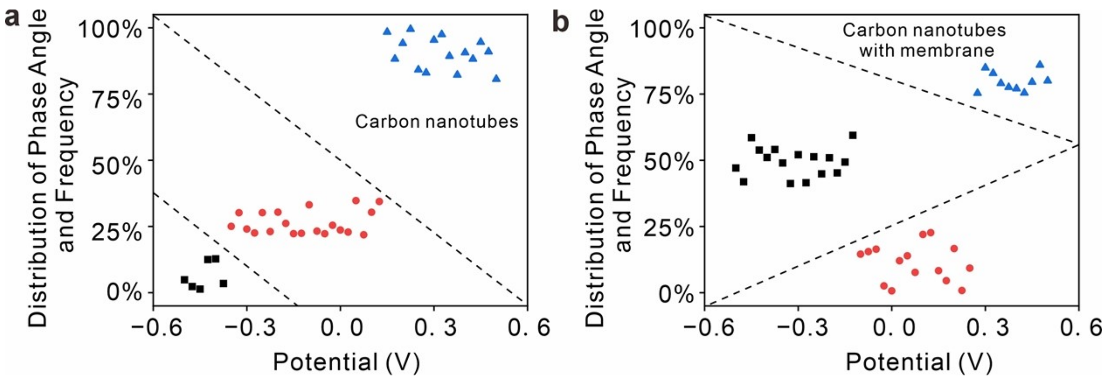

Fig. S13 (a) Classifying data sets through machine learning for carboxylated carbon nanotube without membranes. (b) Classifying data sets through machine learning for carboxylated carbon nanotube with membranes.

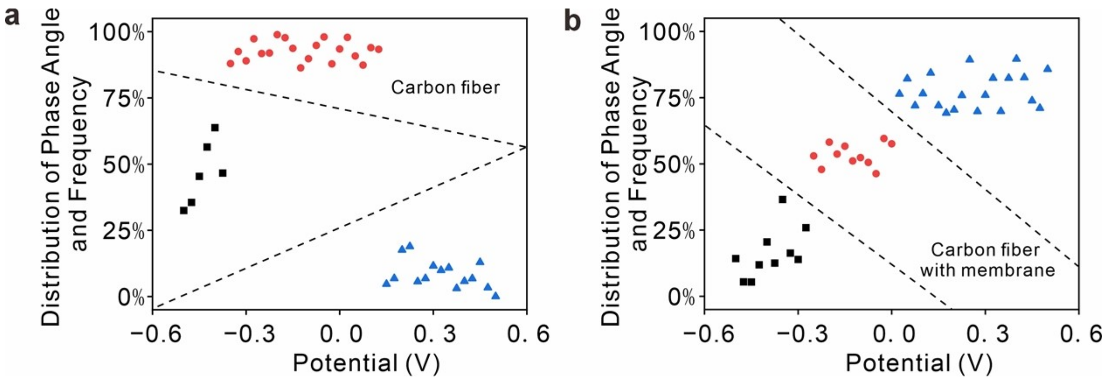

Fig. S14 (a) Classifying data sets through machine learning for carbon fiber without membranes. (b) Classifying data sets through machine learning for carbon fiber with membranes.

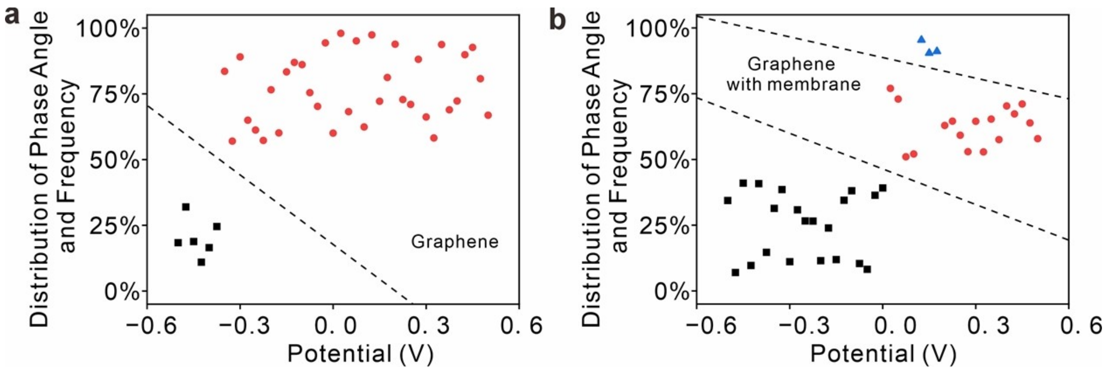

Fig. S15 (a) Classifying data sets through machine learning for graphene without membranes. (b) Classifying data sets through machine learning for graphene with membranes.

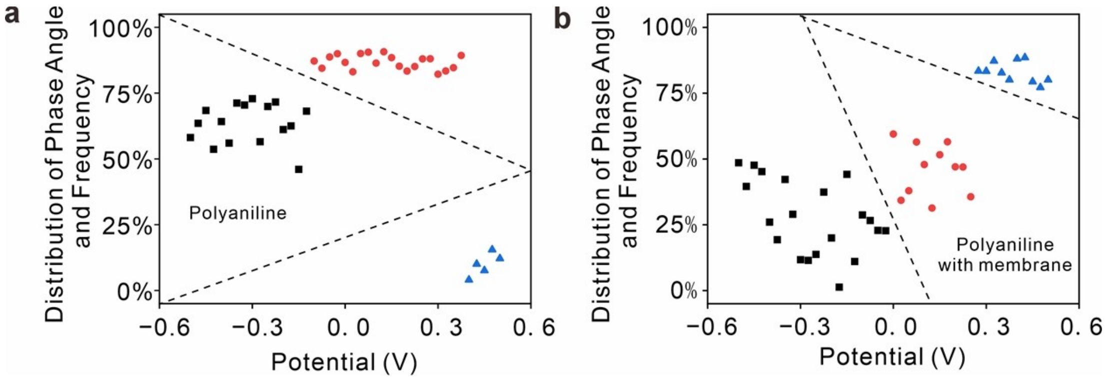

Fig. S16 (a) Classifying data sets through machine learning for polyaniline without membranes. (b) Classifying data sets through machine learning for polyaniline with membranes.

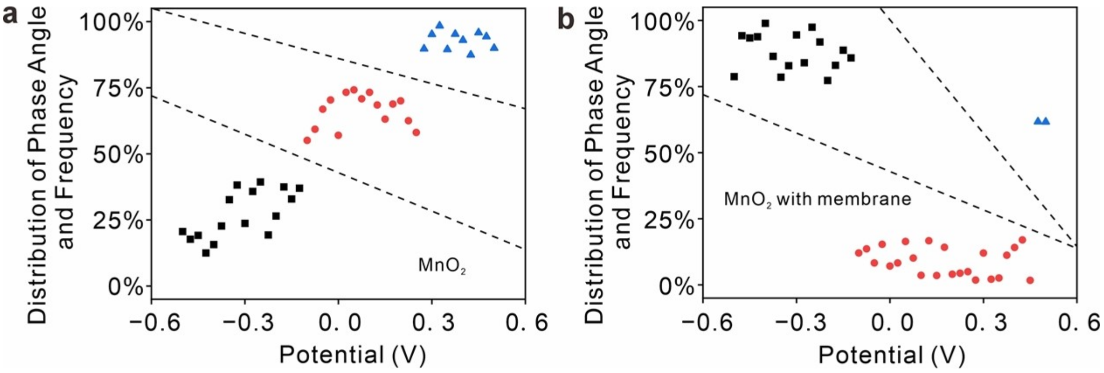

Fig. S17 (a) Classifying data sets through machine learning for MnO_2 without membranes. (b) Classifying data sets through machine learning for MnO_2 with membranes.

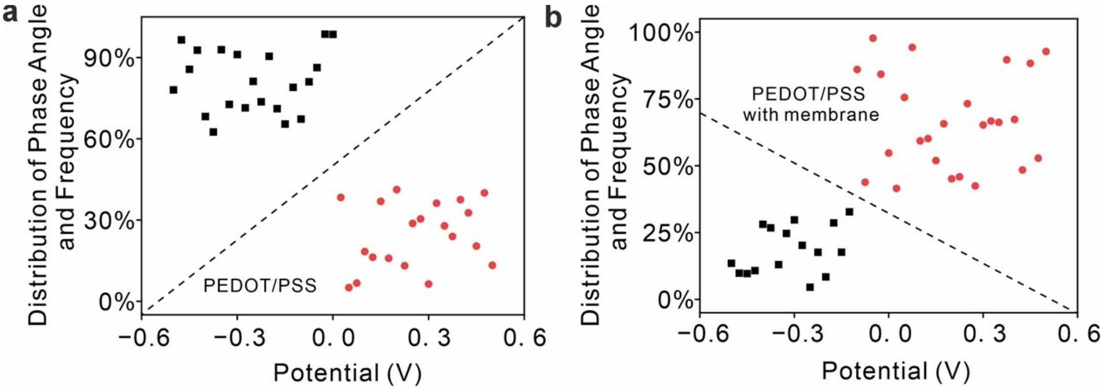

Fig. S18 (a) Classifying data sets through machine learning for PEDOT/PSS without membranes. (b) Classifying data sets through machine learning for PEDOT/PSS with membranes.

1.4 Fig. S19

An example and analysis of the DRT simulation process.

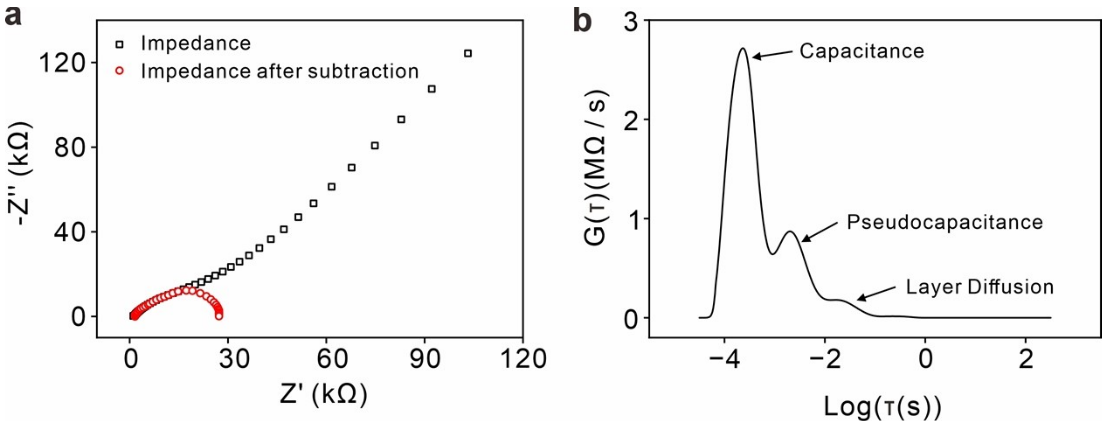

Fig. S19 (a) Impedance data of graphene with the ion-selective membrane and impedance data after subtraction. (b) The distribution of relaxation times (τ) and G(τ) simulated through DRTtools.

Raw impedance data in Fig. S19(a) are presented by black squares, and impedance data after subtraction are red circles. The subtraction implies the elimination of infinite or semi-infinite diffusion processes. Three different peaks are marked as three interfacial processes in Fig. S19(b). Among them, the relaxation time is the shortest for the capacitive process, followed by the pseudocapacitance process, and the slowest is the interlayer diffusion process between graphene sheets.[1] The final interface process is presented in Table S2, including the infinite/semi-infinite diffusion process that has been subtracted.

1.5 Fig. S20

Comparisons in this section were conducted between cyclic voltammetry (CVs) and interfacial processes in membrane-less electrode systems to ensure the accuracy of the methods. Fig. S7a depicts the cyclic voltammograms for six different materials in 0.1 M NaCl solutions, demonstrating their ability to store charge to some degree. Three distinct interfacial processes about charge storage were depicted by different symbols in Fig. S7b, with varying primary processes for different materials. Primary processes for carboxylated carbon nanotube, carbon fiber, and graphene are capacitive processes (I_C processes), while for other materials they are redox processes (I_D processes).

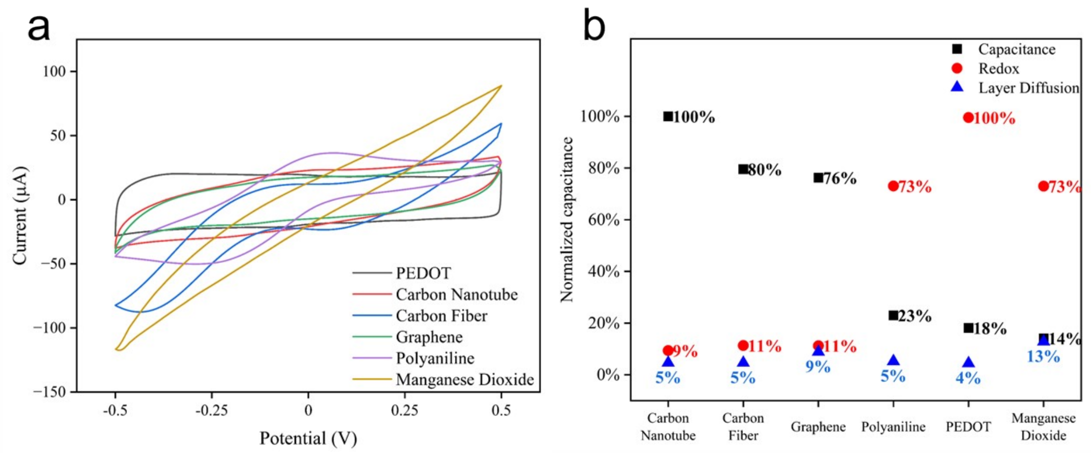

Fig. S20 (a) CVs under 10mV • s^{-1} for six different materials in NaCl solutions. The gray line represents PEDOT/PSS, the red line corresponds to carbon nanotube, the blue line indicates carbon fiber, the green line represents graphene, the purple line is polyaniline and the orange line denotes MnO_2. (b) Normalized capacitance of interfacial processes for six different materials in NaCl solutions. Gray squares represent capacitive processes, red cycles correspond to redox processes and blue triangles denote processes about layer diffusion in materials.

1.6 Fig. S21-S26

Results of SPECSs for materials with symmetric primary charging processes. Fig. S21 is carboxylated carbon nanotube, Fig. S22 is carbon fiber, Fig. S23 is graphene, Fig. S24 is polyaniline, Fig. S25 is MnO_2, and Fig. S26 is PEDOT/PSS.

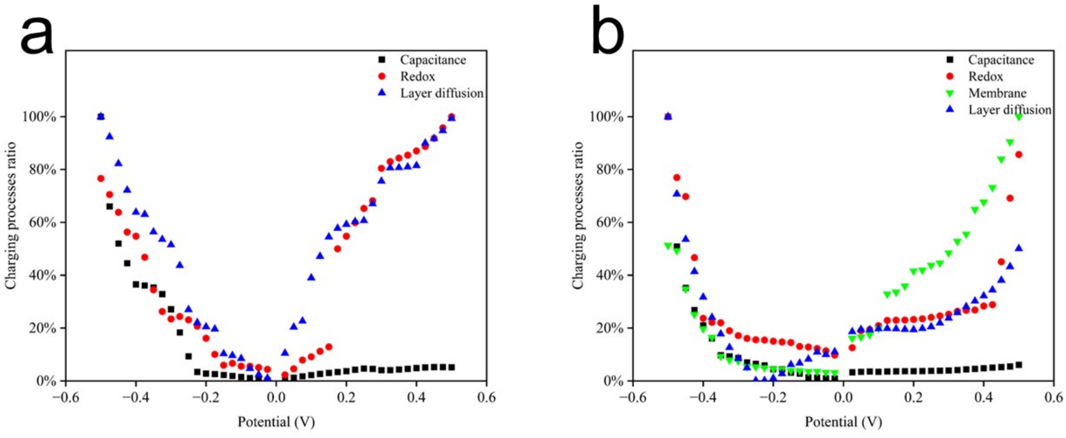

Fig. S21 (a) Results of SPECSs for carboxylated carbon nanotube without membranes. (b) Results of SPECSs for carboxylated carbon nanotube with membranes. Black squares represent capacitive processes, red cycles indicate redox processes, blue triangles processes about layer diffusion in materials and green inverted triangles correspond to membrane processes.

The main charging process of carboxylated carbon nanotube is a capacitive process. The black squares representing capacitance in Fig. S21 demonstrated asymmetry, and charging processes under negative overpotentials were predominant. This could be attributed to the modified functional groups which can attract ions of opposite charge. It can be seen from the comparison between Fig. S21(a) and Fig. S21(b) that the membrane process has no effect on the asymmetric capacitive characteristics of carboxylated carbon nanotube.

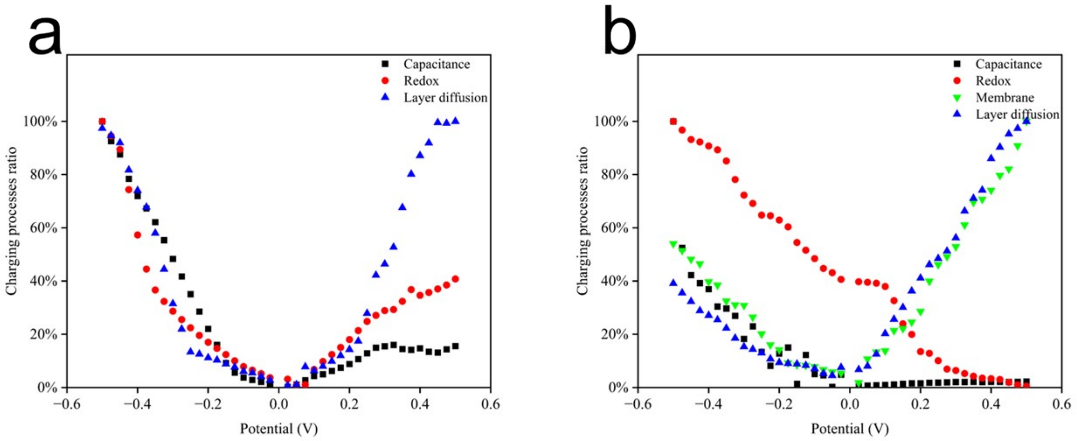

Fig. S22 (a) Results of SPECSs for carbon fiber without membranes. (b) Results of SPECSs for carbon fiber with membranes. Black squares represent capacitive processes, red cycles indicate redox processes, blue triangles processes about layer diffusion in materials and green inverted triangles correspond to membrane processes.

The main charging process of carbon fiber is a capacitive process. The black squares representing capacitance in Fig. S22 demonstrated asymmetry and charging processes under negative overpotentials were predominant. At the same time, the proportion of the capacitive process has increased compared with that of carboxylated carbon nanotube. The membrane process has no effect on the asymmetric capacitive characteristics of carbon fiber but inhibited the capacitive process under positive overpotentials.

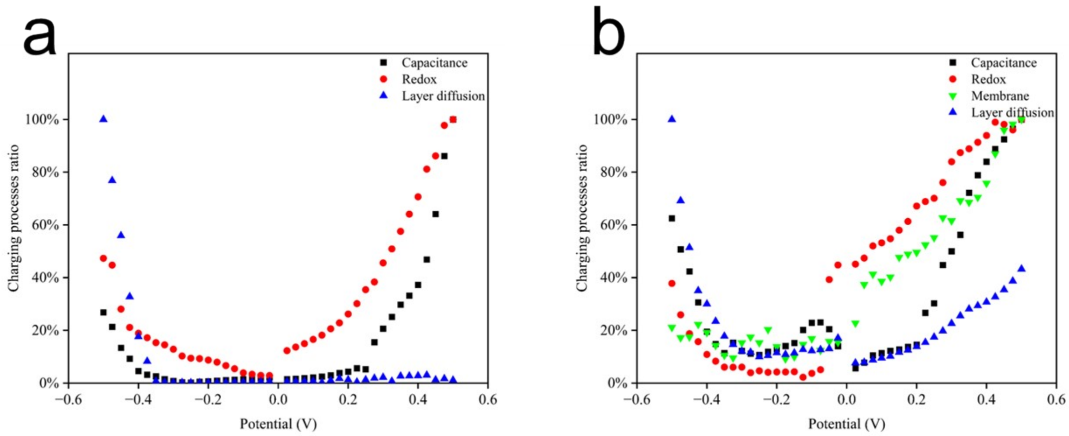

Fig. S23 (a) Results of SPECSs for graphene without membranes. (b) Results of SPECSs for graphene with membranes. Black squares represent capacitive processes, red cycles indicate redox processes, blue triangles processes about layer diffusion in materials and green inverted triangles correspond to membrane processes.

The main charging process of graphene is a capacitive process. The black squares representing capacitance in Fig. S23 demonstrated symmetry. This implies that for capacitive processes, different positive and negative overpotentials do not have a significant effect. This property might be crucial for transduction layers. It can be observed from Fig. S23(b) that the membrane will not influence the symmetric capacitive characteristics of graphene.

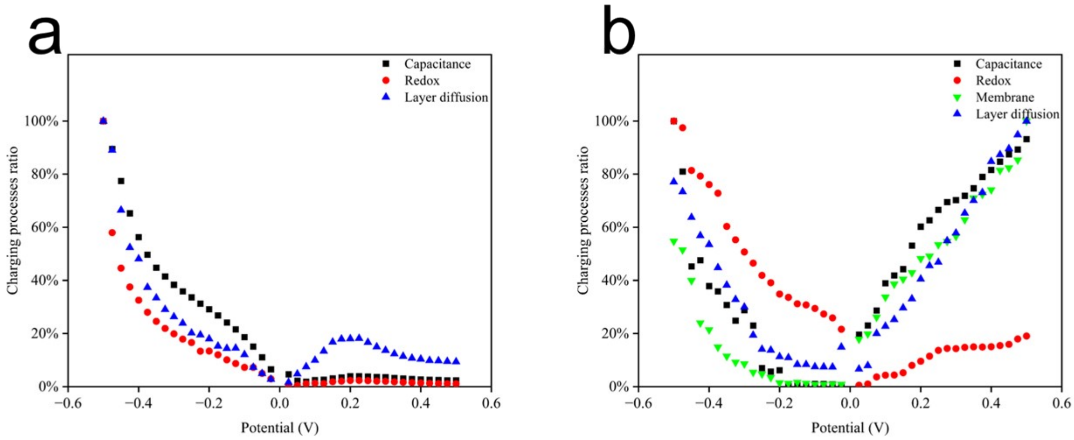

Fig. S24 (a) Results of SPECSs for polyaniline without membranes. (b) Results of SPECSs for polyaniline with membranes. Black squares represent capacitive processes, red cycles indicate redox processes, blue triangles processes about layer diffusion in materials and green inverted triangles correspond to membrane processes.

The main charging process of polyaniline is a redox process. The red circles representing pseudocapacitance in Fig. S24 demonstrated asymmetry, and charging processes under negative overpotentials were predominant. Besides, both capacitances and layer diffusions are also asymmetrical. This can be ascribed to the modified functional groups on the polymer, which has an irregular structure and can attract ions of opposite charge. It can be observed from the comparison between Fig. S24(a) and Fig. S24(b) that the membrane process affected the asymmetric processes of capacitances and layer diffusions, except for pseudocapacitance.

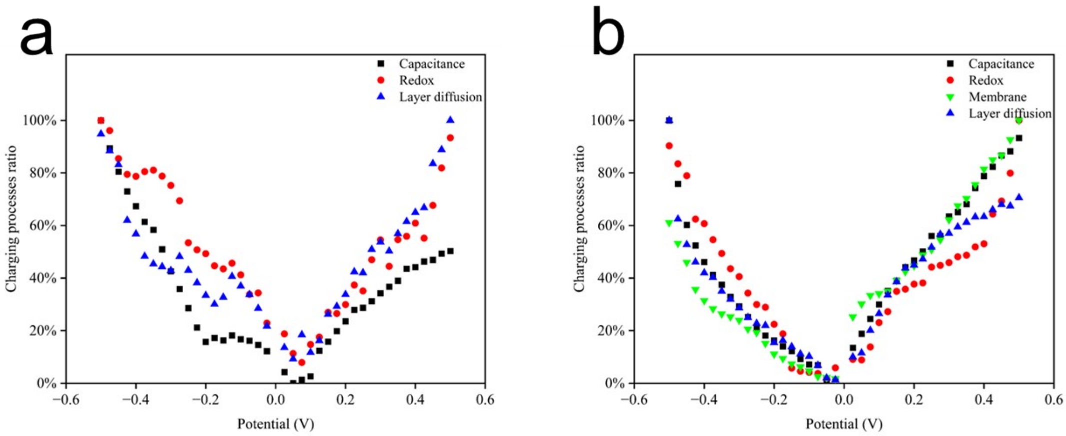

Fig. S25 (a) Results of SPECSs for MnO_2 without membranes. (b) Results of SPECSs for MnO_2 with membranes. Black squares represent capacitive processes, red cycles indicate redox processes, blue triangles processes about layer diffusion in materials and green inverted triangles correspond to membrane processes.

The main charging process of MnO_2 is a redox process. The red circles representing pseudocapacitance in Fig. S25 demonstrated symmetry. This could be attributed to the surface redox reaction. It can be observed from Fig. S25(b) that the membrane will not influence the symmetric pseudocapacitive characteristics of MnO_2.

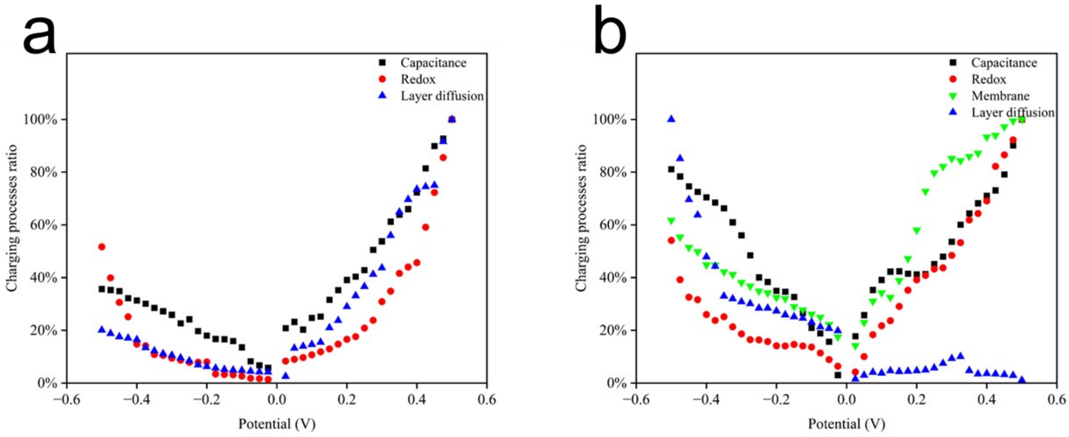

Fig. S26 (a) Results of SPECSs for PEDOT/PSS without membranes. (b) Results of SPECSs for PEDOT/PSS with membranes. Black squares represent capacitive processes, red cycles indicate redox processes, blue triangles processes about layer diffusion in materials and green inverted triangles correspond to membrane processes.

The main charging process of PEDOT/PSS is a redox process. The red circles representing pseudocapacitance in Fig. S26 demonstrated symmetry. Different positive and negative overpotentials could not have a significant effect on the surface redox reaction. It can be observed from Fig. S26(b) that the membrane will not influence the symmetric pseudocapacitive characteristics of PEDOT/PSS.

2. Tables

2.1 ***Table S1****. Classification of characteristic intervals of different materials.*

Materials	Classification A	Classification B	Classification C
Carboxylated carbon nanotube without membranes	[-0.5, -0.375]	(-0.375, 0.125]	(0.125, 0.5]
Carboxylated carbon nanotube with membranes	[-0.5, -0.125]	(-0.125, 0.25]	(0.25, 0.5]
Carbon fiber without membranes	[-0.5, -0.375]	(-0.375, 0.125]	(0.125, 0.5]
Carbon fiber with membranes	[-0.5, -0.275]	(-0.275, 0]	(0, 0.5]
Graphene without membranes	[-0.5, -0.375]	(-0.375, 5]	/
Graphene with membranes	[-0.5, 0]	(0, 0.1] ∪ [0.2, 0.5]	(0.125, 0.175]
Polyaniline without membranes	[-0.5, -0.125]	(-0.125, 0.375]	(0.375, 0.5]
Polyaniline with membranes	[-0.5, -0.025]	(-0.025, 0.25]	(0.25, 0.5]
MnO_2 without membranes	[-0.5, -0.125]	(-0.125, 0.25]	(0.25, 0.5]
MnO_2 with membranes	[-0.5, -0.125]	(-0.125, 0.45]	(0.45, 0.5]
PEDOT/PSS without membranes	[-0.5, 0]	(0, 0.5]	/
PEDOT/PSS with membranes	[-0.5, -0.125]	(-0.125, 0.5]	/

*2.2 **Table S2**. Interfacial models.*

Materials	Model
Carboxylated carbon nanotube without membranes	$\frac{\Delta E}{R_{S1}} e^{\frac{-t}{R_{S1}C_{D1}}} + \frac{\Delta E}{R_{S2}} e^{\frac{-t}{R_{S2}C_{D2}}} + \sum_{i=1}^{\infty} K_1^i t^{-\frac{1}{2}} + i_R$
Carboxylated carbon nanotube with membranes	$\frac{\Delta E}{R_{S1}} e^{\frac{-t}{R_{S1}C_{D1}}} + \frac{\Delta E}{R_{S2}} e^{\frac{-t}{R_{S2}C_{D2}}} + \sum_{i=1}^{\infty} K_1^i t^{-\frac{1}{2}} + \sum_{m=1}^{\infty} K_2^m \sum_{k=1}^{\infty} e^{K_3^k t} + i_R$
Carbon fiber without membranes	$\frac{\Delta E}{R_{S1}} e^{\frac{-t}{R_{S1}C_{D1}}} + \frac{\Delta E}{R_{S2}} e^{\frac{-t}{R_{S2}C_{D2}}} + \sum_{i=1}^{\infty} K_1^i t^{-\frac{1}{2}} + i_R$
Carbon fiber with membranes	$\frac{\Delta E}{R_{S1}} e^{\frac{-t}{R_{S1}C_{D1}}} + \frac{\Delta E}{R_{S2}} e^{\frac{-t}{R_{S2}C_{D2}}} + \sum_{i=1}^{\infty} K_1^i t^{-\frac{1}{2}} + \sum_{m=1}^{\infty} K_2^m \sum_{k=1}^{\infty} e^{K_3^k t} + i_R$
Graphene without membranes	$\frac{\Delta E}{R_{S1}} e^{\frac{-t}{R_{S1}C_{D1}}} + \frac{\Delta E}{R_{S2}} e^{\frac{-t}{R_{S2}C_{D2}}} + \sum_{i=1}^{\infty} K_1^i t^{-\frac{1}{2}} + \sum_{j=1}^{\infty} K_2^j \sum_{k=1}^{\infty} e^{K_3^k t} + i_R$
Graphene with membranes	$\frac{\Delta E}{R_{S1}} e^{\frac{-t}{R_{S1}C_{D1}}} + \frac{\Delta E}{R_{S2}} e^{\frac{-t}{R_{S2}C_{D2}}} + \sum_{i=1}^{\infty} K_1^i t^{-\frac{1}{2}}$ $+ \sum_{j=1}^{\infty} K_2^j \sum_{k=1}^{\infty} e^{K_3^k t} + \sum_{m=1}^{\infty} K_2^m \sum_{k=1}^{\infty} e^{K_3^k t} + i_R$
Polyaniline without membranes	$\sum_{i=1}^{\infty} \frac{\Delta E}{R_{Si}} e^{\frac{-t}{R_{Si}C_{Di}}} +$ $\sum_{i=1}^{\infty} K_1^i t^{-\frac{1}{2}} + \sum_{j=1}^{\infty} K_1^j t^{-\frac{1}{2}} + \sum_{m=1}^{\infty} K_2^m \sum_{k=1}^{\infty} e^{K_3^k t} + \sum_{n=1}^{\infty} K_2^n \sum_{k=1}^{\infty} e^{K_3^k t} + i_R$
Polyaniline with membranes	$\sum_{i=1}^{\infty} \frac{\Delta E}{R_{Si}} e^{\frac{-t}{R_{Si}C_{Di}}} +$

	$\sum_{i=1}^{\infty} K_1^i t^{-\frac{1}{2}} + \sum_{j=1}^{\infty} K_1^j t^{-\frac{1}{2}} + \sum_{m=1}^{\infty} K_2^m \sum_{k=1}^{\infty} e^{K_3^k t} + \sum_{n=1}^{\infty} K_2^n \sum_{k=1}^{\infty} e^{K_3^k t} + \sum_{t=1}^{\infty} K_2^t \sum_{k=1}^{\infty} e^{K_3^k t} + i_R$
MnO_2 without membranes	$\frac{\Delta E}{R_{S1}} e^{\frac{-t}{R_{S1}C_{D1}}} + \sum_{i=1}^{\infty} K_1^i t^{-\frac{1}{2}} + \sum_{j=1}^{\infty} K_1^j t^{-\frac{1}{2}} + \sum_{m=1}^{\infty} K_2^m \sum_{k=1}^{\infty} e^{K_3^k t} + \sum_{n=1}^{\infty} K_2^n \sum_{k=1}^{\infty} e^{K_3^k t} + i_R$
MnO_2 with membranes	$\frac{\Delta E}{R_{S1}} e^{\frac{-t}{R_{S1}C_{D1}}} + \sum_{i=1}^{\infty} K_1^i t^{-\frac{1}{2}} + \sum_{j=1}^{\infty} K_1^j t^{-\frac{1}{2}} + \sum_{m=1}^{\infty} K_2^m \sum_{k=1}^{\infty} e^{K_3^k t} + \sum_{n=1}^{\infty} K_2^n \sum_{k=1}^{\infty} e^{K_3^k t} + i_R$
PEDOT/PSS without membranes	$\sum_{i=1}^{\infty} \frac{\Delta E}{R_{Si}} e^{\frac{-t}{R_{Si}C_{Di}}} + \sum_{i=1}^{\infty} K_1^i t^{-\frac{1}{2}} + \sum_{j=1}^{\infty} K_1^j t^{-\frac{1}{2}} + \sum_{m=1}^{\infty} K_2^m \sum_{k=1}^{\infty} e^{K_3^k t} + \sum_{n=1}^{\infty} K_2^n \sum_{k=1}^{\infty} e^{K_3^k t} + \sum_{t=1}^{\infty} K_2^t \sum_{k=1}^{\infty} e^{K_3^k t} + i_R$
PEDOT/PSS with membranes	$\sum_{i=1}^{\infty} \frac{\Delta E}{R_{Si}} e^{\frac{-t}{R_{Si}C_{Di}}} + \sum_{i=1}^{\infty} K_1^i t^{-\frac{1}{2}} + \sum_{j=1}^{\infty} K_1^j t^{-\frac{1}{2}} + \sum_{m=1}^{\infty} K_2^m \sum_{k=1}^{\infty} e^{K_3^k t} + \sum_{n=1}^{\infty} K_2^n \sum_{k=1}^{\infty} e^{K_3^k t} + \sum_{t=1}^{\infty} K_2^t \sum_{k=1}^{\infty} e^{K_3^k t} + i_R$

2.3 ***Table S3****. Parameters in Interfacial models.*

Parameter	Meaning	Equation
C_1	Real components of capacitance	$C_1 = \frac{-Z''(\omega)}{\omega\lvert Z(\omega)\rvert^2}$
C_2	Imaginary components of capacitance	$C_2 = \frac{Z'(\omega)}{\omega\lvert Z(\omega)\rvert^2}$
K_1	Constant of the semi-infinite diffusion condition	$K_1 = \frac{nFA\sqrt{D}C}{\sqrt{\pi}}$
K_2	Constants of the finite diffusion condition	$K_2 = \frac{4nFADC}{l}$
K_3		$K_3 = \frac{-(2n-1)^2\pi^2 D}{l^2}$

where ω (unit: Hz) is the applied frequency, Z(ω), Z'(ω) and Z"(ω) are impendences which are all functions of ω, F (96485 $C\cdot mol^{-1}$) is the Faraday's constant, D (unit: $cm^2\cdot s^{-1}$) is the diffusion coefficient, A (unit: cm^2) is the geometric area of the electrode, C (unit: $mol\cdot cm^{-3}$) is the concentration of target species, n is the number of charges transferred.

3. Experimental section

3.1 Cleaning processes

Firstly, the working electrode should be polished by three types of Al_2O_3 (1, 0.3, and 0.05 μm) for about 1-2 minutes. After burnished, the electrode should be immersed in respectively HNO_3, C_2H_5OH and H_2O under a hyper-acoustic condition for at least 30 s. Then materials (solid contacts or solid contacts and membranes) would be dipped on the electrode after the electrode drying.

3.2 Information about different materials and membranes

Materials in this work including: PEDOT/PSS, carbon fiber, carboxylated carbon nanotube, polyaniline, and MnO_2. Each of the reagents purchased from Sinopharm Chemical Reagent Co., Ltd. (China) was analytical grade. The bulk solution at pH = 7 consisted of 0.1 M NaCl.

For electrodes modified with different transduction layers: for each of the six distinct transduction materials, 10 mg was dispersed in 1 mL of THF and subjected to ultrasonication for 1 hour to ensure uniform dispersion. Subsequently, 10 μL of the well-dispersed liquid was pipetted and drop-cast onto the surface of a cleaned bare GCE, followed by complete drying in a fume hood to obtain electrodes modified with the transduction layer materials.

For the Na^+-selective membrane and all-solid-state ion-selective electrodes: 1wt% sodium ionophore (ETH 1062), 1.02 wt% NaTFPB, 65.32 wt% *o*-NPOE, and 32.66 wt% PVC are 300 mg in total and dissolved in 3 mL THF to prepare the membrane solution. Then uniformly coat 20 μL of the cationic membrane solution onto electrode surfaces with different transduction materials to obtain a sodium ion all-solid-state ion-selective electrode (Na^{2+}-SCISE).

3.3 Parameters of electrochemical experiments

Five different scan rates (mV/s) were employed in the cyclic voltammograms, with

each scan rate being repeated five times, specifically 1, 10, 25, 50, and 100 $mV \cdot s^{-1}$. When performing SPECS, the polarization time for signal recording was set to 100 s, with a relaxation interval time of 1000 s. After completing these two processes, a new cycle will be initiated with a voltage step of 0.025 V, and the sampling interval is configured to 0.01 s. The initial and final frequencies used for EIS were 10000 Hz and 0.01 Hz, respectively. Prior to conducting an impedance test, it is essential to apply a constant potential of 300 seconds to the system for equilibrium. After each EIS test, the applied potentiostat requires a potential step of 0.025 V, and the relaxation time after each EIS cycle is set to 1000 s.

References

[1] F. Ciucci, Modeling electrochemical impedance spectroscopy, Curr. Opin. Electrochem., 13 (2019) 132–139.
